# A robust deep learning detector for sleep spindles and K-complexes: towards population norms

**DOI:** 10.1038/s41598-023-50736-7

**Published:** 2024-01-02

**Authors:** Nicolás I. Tapia-Rivas, Pablo A. Estévez, José A. Cortes-Briones

**Affiliations:** 1 Department of Electrical Engineering, University of Chile, Santiago, Chile; 2 Millennium Institute of Intelligent Healthcare Engineering, Santiago, Chile; 3 IMPACT, Center of Interventional Medicine for Precision and Advanced Cellular Therapy, Santiago, Chile; 4 Schizophrenia and Neuropharmacology Research Group at Yale (SNRGY), Department of Psychiatry, Yale University School of Medicine, New Haven, CT USA; 5 Abraham Ribicoff Research Facilities, Connecticut Mental Health Center, New Haven, CT USA; 6 VA Connecticut Healthcare System, West Haven, CT USA

**Keywords:** Neurology, Computational biology and bioinformatics, Machine learning

## Abstract

Sleep spindles (SSs) and K-complexes (KCs) are brain patterns involved in cognitive functions that appear during sleep. Large-scale sleep studies would benefit from precise and robust automatic sleep event detectors, capable of adapting the variability in both electroencephalography (EEG) signals and expert annotation rules. We introduce the Sleep EEG Event Detector (SEED), a deep learning system that outperforms existing approaches in SS and KC detection, reaching an F1-score of 80.5% and 83.7%, respectively, on the MASS2 dataset. SEED transfers well and requires minimal fine-tuning for new datasets and annotation styles. Remarkably, SEED substantially reduces the required amount of annotated data by using a novel pretraining approach that leverages the rule-based detector A7. An analysis of 11,224 subjects revealed that SEED's detections provide better estimates of SS population statistics than existing approaches. SEED is a powerful resource for obtaining sleep-event statistics that could be useful for establishing population norms.

## Introduction

**Figure 1:**
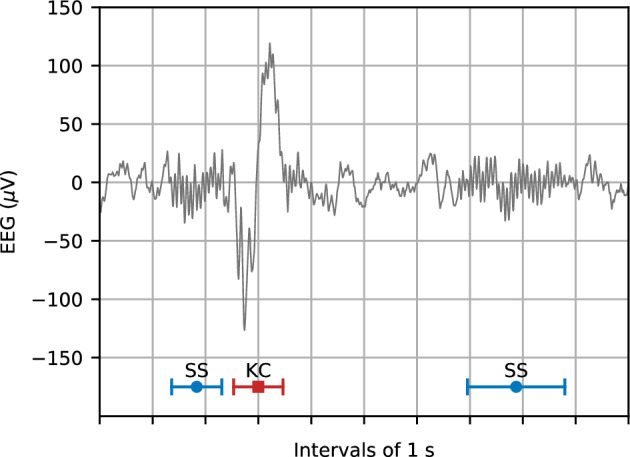
An EEG segment during sleep stage N2. One K-complex (KC) and two sleep spindles (SSs) are shown as annotated by a human expert.

Sleep has a central role in cognitive function^1^, brain development^2^, and neurological and neuropsychiatric disorders^3,4^. During sleep, the brain transitions through five stages: wakefulness (W), rapid eye movement sleep (REM), and three non-REM sleep stages (N1, N2, and N3)^5^. Sleep spindles (SSs) and K-complexes (KCs) are two short-lived neural activity patterns occurring during stage N2 that are typically captured using electroencephalography (EEG) (see Fig. 1). Both patterns are involved in several cognitive functions including memory, learning, and stimulus processing^6,7^.

Alterations in the duration, amplitude, frequency, and density of SSs have been associated with neuropsychiatric and neurological disorders. For example, recent studies suggest that SS alterations could be biomarkers of focal epilepsy^8^, autism spectrum disorder^9^, and clinical high-risk psychosis^3^. Likewise, KC alterations have been associated with sleep apnea^10^ and Alzheimer’s disease^4^.

While promising, these studies lack the sample sizes required to assess the clinical potential of SS or KC and validate them as biomarkers. One of the main obstacles to increasing sample size is the reliance on costly expert annotations. Furthermore, expert annotations have low inter-rater reliability^11^, thus a consensus of experts is required to achieve high-quality annotations^11,12^. For this reason, automatic SS and KC detection is an active area of research aiming to overcome the limitations of manual detection while maintaining high accuracy and adaptability to diverse EEG signals.

While alterations in SS parameters may indicate illness, there is also considerable variation within the normal population related to factors like sex, age, and origin^13^. A one-size-fits-all approach is unlikely to be effective in developing SS-based diagnostic strategies. Instead, abnormal deviation detection needs a reference of healthy ranges across demographics. Large-scale studies with automatic detectors can provide such characterization. For example, Purcell et al^14^*.* studied variation across sex, age, and race, starting from the age of 5, while more recently, Kwon et al^15^*.* examined variation from birth to the age of 18. The main limitation of these studies is the detection accuracy. They could be improved by detectors that are not only more accurate but also robust to be trusted in a wide variety of demographics.

Several methods have been proposed for the automatic detection of SSs and KCs. These approaches use information obtained from signals in the time^16–20^ and/or frequency domains^21–28^. Traditionally, automatic SS and KC detection algorithms such as A7 for SSs^21^, and Spinky for SSs and KCs^25^, rely on expert-informed handcrafted features extracted from EEG signals. The main advantage of these rule-based detectors is that predictions depend on familiar and easy-to-understand criteria. However, according to the literature, the definitions of SSs and KCs are vague and incomplete^7,23^, e.g., some consider that the 0.5-s minimum duration for spindles is arbitrary^11^. This makes it difficult to pre-define versatile-enough, one-size-fits-all features, and rules to suit most SS and KC definitions. Recently, deep learning detectors, a set of algorithms that automatically extract and learn features from the raw data, have been shown to outperform rule-based, handcrafted-feature algorithms and achieve state-of-the-art performance^16,17,22,23^. However, we identified two main gaps that we address to make deep learning detection of SSs and KCs more accurate and widely adopted by sleep researchers.

First, we hypothesized that better performance could be achieved by better context processing. Although SS and KC duration is ~ 1 s, experts typically analyze larger segments, usually ~ 25s^11^. In this way, experts contextualize the pattern in the surrounding activity to deal with noise, adapt to subject-level variation, and precisely determine the pattern’s onset and ending times. That is, experts leverage temporal dynamics in the EEG that are relevant for detection.

In the deep learning literature, recurrent neural networks (RNNs) are recommended as the method of choice to model temporal dynamics in time series such as EEG signals, since they are specifically designed for sequence processing. However, even with algorithmic improvements, recurrent architectures struggle with long sequences because they forget^29,30^: they cannot retrieve and harness information from distant contexts. In practice, a length of 250–500 samples is usually regarded as a safe maximum. For this reason, convolutional neural networks (CNNs) are usually used as a previous stage of RNNs. Several CNN + RNN models have been developed for sleep stage scoring, where EEG segments of 30 s are processed^31,32^.

Typically, sleep EEG events such as SSs and KCs have a duration of approximately one second. In the literature, CNN + RNN methods for detecting sleep EEG events use short EEG segments as inputs (~ 1 s), i.e., segments with less than 500 samples^22,33^. Some CNN-based models process 20-s segments, thus using a longer context^16,17^.

In order to use 20-s contexts for detecting SS and KC events, we propose a DL-based architecture that combines CNNs and RNNs. Convolutional architectures excel at extracting local features and reducing input width while keeping relevant information. Therefore, a convolutional stage can be used to transform a 20 s EEG segment into a shorter time series, where each sample already encodes local signal patterns (around 1 s of signal). Then, a recurrent stage can be used to model this shorter, richer sequence. We conducted a preliminary exploration of sequential context processing in a previous work^34^.

The second gap is that the aforementioned SS and KC detectors have not been thoroughly validated on large-scale datasets, for their correct behavior and generalization capabilities. Such validation is important for human raters to understand their operation limits and to adopt them as a replacement for well-known rule-based detectors. To address this gap, we conducted an extensive quantitative and qualitative validation of the proposed detection method, using both labeled and large unlabeled datasets. Moreover, we build upon previous research and provide improved ranges of SS parameters across demographics.

In summary, we introduce and validate the Sleep EEG Event Detector (SEED), a novel end-to-end deep learning approach to detecting SS and KC in sleep EEG signals. This work has 4 main contributions: First, a detailed description of SEED’s training and architecture, which uses convolutional layers for local-feature extraction, and recurrent layers for modeling long-term (20 s) signal dynamics; second, an extensive quantitative and qualitative validation assessment of SEED using 2 labeled and 7 unlabeled datasets comprising a total of 11,499 subjects; third, an assessment of SEED’s generalizability through transfer learning experiments; and fourth, an estimation of SS population statistics using SEED’s state-of-the-art SS detection performance. These results are a significant advancement over existing large-scale SS population statistics, which are based on a less-accurate, previous-generation detectors.

## Results

### Datasets overview

The data were extracted from 9 sleep EEG datasets and comprised 11,499 single-channel EEG recordings collected during stage N2 from channels C3 or C4. Two datasets, MASS2 and MODA, have expert annotations for sleep stage, SSs and/or KCs. The remaining datasets, CAP and NSRR6 (which comprises 6 datasets), have expert annotations for sleep stage only (see Table 1). In what follows, MASS2 and MODA will be termed labeled datasets, and CAP and NSRR6 will be termed unlabeled datasets.

**Table 1:** Description of sleep EEG datasets.

	Labeled datasets				Unlabeled datasets	
	MASS2-SS-E1	MASS2-SS-E2	MASS2-KC	MODA	CAP	NSRR6
Subjects	15	15	15	180	80	11,224
Age (mean ± SD)	23.6 ± 3.7	23.6 ± 3.7	23.6 ± 3.7	40.6 ± 19.4	39.5 ± 16.9	58.6 ± 23.5
Sampling rate (Hz)	256	256	256	256	100–512	125–512
Annotated event	SS	SS	KC	SS	N.A	N.A
Annotated or selected segments	Stage N2	Stage N2	Stage N2	115 s segments*	Stage N2	Stage N2
Annotated or selected size (h)	60.01	60.01	60.01	24.97	251.64	36,548.1
Annotation source	One expert	One expert	One expert	Consensus of 31–42 exp	N.A	N.A
Total events	9,990	21,846	8,781	5,272	N.A	N.A
Density (epm)	2.72	6.02	2.49	3.52	N.A	N.A
Mean duration (s)	0.83	1.20	0.73	0.84	N.A	N.A

SS: sleep spindle; KC: K-complex; N.A.: not applicable; epm: events per minute. NSRR6 combines the datasets CHAT, CCSHS, CFS, SHHS, MrOS and SOF. Dataset details can be found in the main text and the Methods section. The terms labeled and unlabeled refer to the availability of event (SS or KC) annotations since every dataset has sleep stage annotations. * Segments of 115 s were randomly extracted from sleep stage N2.

#### Labeled datasets

MASS2^35^ comprises 3 datasets: (1) SSs annotated by expert E1 (MASS2-SS-E1) and (2) expert E2 (MASS2-SS-E2), and (3) KCs annotated by expert E1 (MASS2-KC); MODA^12^ comprises data collected from 2 age groups: (1) young (mean age of 24.1 years old) and (2) older adults (mean age of 62.0 years old). These datasets were used for performance analyses.

#### Unlabeled datasets

Following Purcell et al^14^*.*, NSRR6 comprises 6 sleep datasets from the National Sleep Research Resource (NSRR)^36^: CHAT^37^, CCSHS^38^, CFS^39^, SHHS^40^, MrOS^41^, and SOF^42^. The NSRR6 dataset was used for population statistics analysis. Lastly, the CAP dataset^43,44^ was used for transfer learning analyses (for details on all datasets, see Methods).

## Sleep EEG event detector (SEED)

**Figure 2:**
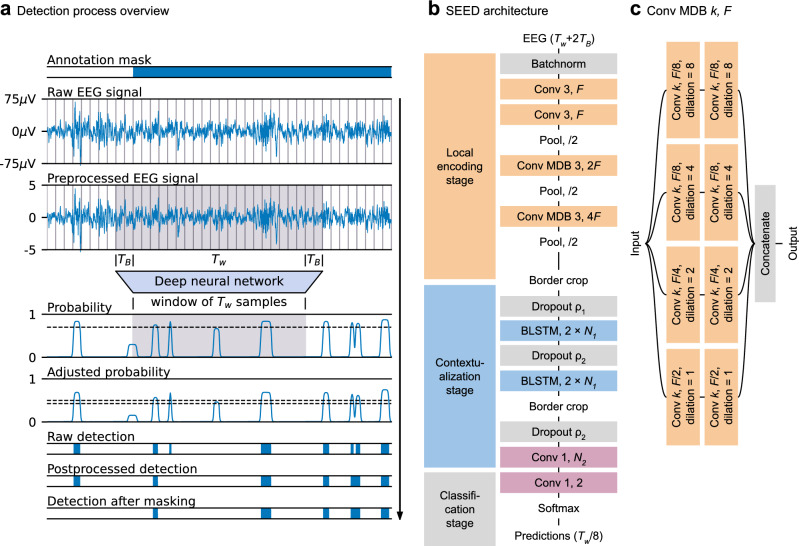
SEED’s event detection process and architecture. (**a**) Raw EEG signals are preprocessed and segmented in windows of ${T}_{w}$=20 s samples. Each window is fed to the deep neural network underlying SEED, which estimates each sample’s probability of being part of an event (SS or KC). To avoid border effects, signal segments of ${T}_{B}$ samples are concatenated to both input window’s borders, which are dropped afterward. The raw probabilities are adjusted and thresholded using a pair of thresholds, one for detection and another for duration estimation. The resulting events can be post-processed using expert knowledge. Finally, events outside the valid annotation mask (e.g., N2 stages) are discarded. (**b**) High-level description of SEED's neural network architecture. (**c**) Definition of Convolutional Multi-dilated Block (Conv MDB) k, F. It has the same number of parameters as a sequence of two Conv k, F layers, but with a larger receptive field.

For an EEG signal segment, the proposed Sleep EEG Event Detector (SEED) estimates each sample’s probability of being part of an event of interest (see Fig. 2a). SEED’s estimation is obtained by combining a convolutional neural network to extract local features and a recurrent neural network to integrate local features within a wide temporal context of 20 s (see Fig. 2b). Each event’s onset and ending point are obtained by thresholding SEED’s estimated probabilities.

### Baselines

Four state-of-the-art deep learning sleep event detectors were used for baseline comparisons: DOSED^16^ was used for SS and KC detection, SpindleNet^22^ was used for SS detection, SpindleU-Net^17^ was used for SS detection, and DKL-KC^23^ was used for KC detection. In addition, two representative rule-based, handcrafted feature detectors were used for comparisons: A7^21^ was used for SS detection, and Spinky^25^ was used for KC detection. DOSED, A7, and Spinky have an open-source training implementation that allowed us to assess them using the same training settings that were used for SEED. No open-source implementations were available for the other detectors; thus, comparisons were conducted using published reports of their performance.

### Performance comparison

SEED’s and the baseline detectors’ performance are reported in Table 2. Following Warby et al*.*’s work^11^ using Intersection over Union (IoU) for obtaining detection metrics, we computed recall, precision, and F1-score. In addition, we used mean IoU (mIoU) to capture localization accuracy, i.e., the accuracy at predicting the onset and ending points of events. Since recordings from the MASS2 dataset influenced hyperparameter and architectural decisions during SEED's development, we conducted an additional assessment of SEED’s performance on a subset of subjects from the MASS2 dataset that was held out and not used during SEED’s development. The results obtained in this subset were similar to the results reported in Table 2, indicating negligible design overfitting (see Supplementary Table 1).

**Table 2:** SS and KC detection performance.

Dataset	Detector	F1-score (%)		mIoU (%)	
		Mean ± SD	*p*-value	Mean ± SD	*p*-value
MASS2-SS-E1 (15 subjects)	SEED	80.8 ± 2.1		84.8 ± 1.2	
	DOSED	76.8 ± 2.9	< 0.001	74.7 ± 2.1	< 0.001
	A7	73.0 ± 3.4	< 0.001	73.9 ± 1.0	< 0.001
MASS2-SS-E1 (19 subjects)	SEED	80.5 ± 2.1		84.7 ± 1.0	
	SpindleU-Net	80.3 ± 1.9*	0.848	73.5	-
MASS2-SS-E2 (15 subjects)	SEED	86.1 ± 2.0		78.7 ± 1.1	
	DOSED	82.5 ± 2.5	< 0.001	73.1 ± 1.1	< 0.001
	A7	74.9 ± 2.8	< 0.001	74.7 ± 1.1	< 0.001
	SpindleU-Net	85.4 ± 2.7*	0.615	N.A	-
	SpindleNet	83.0 ± 2.0	0.020	N.A	-
MODA	SEED	81.8 ± 1.4		83.4 ± 0.5	
	DOSED	77.5 ± 1.7	< 0.001	71.4 ± 1.1	< 0.001
	A7	73.3 ± 1.9	< 0.001	71.0 ± 0.9	< 0.001
MASS2-KC (15 subjects)	SEED	83.7 ± 1.5		90.6 ± 0.6	
	DOSED	78.1 ± 2.2	< 0.001	72.3 ± 1.4	< 0.001
	Spinky	63.1 ± 3.8	< 0.001	41.2 ± 1.6	< 0.001
MASS2-KC (19 subjects)	SEED	83.6 ± 1.7		90.4 ± 0.4	
	DKL-KC	78.0 ± 2.0	< 0.001	N.A	-

mIoU: mean Intersection over Union; N.A.: not available. Metrics of SEED (proposed detector), DOSED, A7 and Spinky were obtained using open-source implementations, whereas metrics of SpindleU-Net, SpindleNet and DKL-KC were obtained from their original publications. *P*-values are defined against SEED’s performance. * These standard deviations between partitions are not reported in the original publication; these estimations are based on the reported by-subject F1-score (see Methods).

SEED outperformed baseline algorithms in all comparisons on mIoU (when available). Additionally, SEED outperformed baselines on F1-score except for one match in which SpindleU-Net’s and SEED’s F1-scores were not significantly different (*p* = 0.85 in MASS2-SS-E1 and *p* = 0.62 in MASS2-SS-E2). Importantly, SpindleU-Net’s mIoU was 11.2% lower than SEED’s.

The between-subject variability of SEED’s performance was measured with the coefficient of variation (ratio between the standard deviation and the mean) of the F1-score (Table 3). In this metric, SEED outperformed all the baseline detectors. In general, metric variability was higher for SS compared to KC detection.

**Table 3:** Detection performance variability across subjects.

Dataset	Detector	Coefficient of variation (%)	
		Mean ± SD	*p*-value
MASS2-SS-E1	SEED	4.6 ± 0.4	
	DOSED	6.9 ± 0.4	< 0.001
	A7	6.9 ± 0.1	< 0.001
MASS2-SS-E2	SEED	4.0 ± 0.1	
	DOSED	5.2 ± 0.3	< 0.001
	A7	5.8 ± 0.1*	< 0.001
MODA	SEED	9.2 ± 0.4	
	DOSED	11.6 ± 0.5	< 0.001
	A7	16.9 ± 0.4*	< 0.001
MASS2-KC	SEED	3.9 ± 0.2	
	DOSED	5.2 ± 0.3	< 0.001
	Spinky	11.5 ± 0.1	< 0.001

The variability (less is better) is measured as the coefficient of variation of the F1-score. The coefficient of variation is the ratio between the standard deviation and the mean, expressed as a percentage. Due to repeated cross-validation, each subject has 3 available test performance values, allowing many possible coefficients of variations to be obtained by randomly selecting one value per subject. Leveraging that, its distribution was estimated by repeating such random selection 100 times. P-values are defined against SEED’s performance.

* Data with non-normal distribution. See Methods/Statistics for details on the statistical tests.

Following previous work^11^, Table 4 reports the correlations and mean differences between expert rater and detector detections in global event characteristics (scatter plots shown in Supplementary Figs. 1 and 2). The results show that SEED outperformed or matched other detectors’ best performance. SEED’s largest performance improvement was in SS and KC duration estimation. In comparison, DOSED showed a large positive bias in mean event duration, incorrectly overestimating SSs in an average of 0.18 s and KCs in an average of 0.14 s.

**Table 4:** Correlation between experts and detectors for subject-level parameters of SSs and KCs.

Parameter	Detector	R-squared	Mean difference
Mean SS duration (s) (MODA)	SEED	0.62	− 0.017
	DOSED	0.47	0.179
	A7	0.35	− 0.020
SS density (epm) (MODA)	SEED	0.94	0.136
	DOSED	0.90	− 0.154
	A7	0.88	0.177
Mean SS PP amplitude (μV) (MODA)	SEED	0.99	0.815
	DOSED	0.98	1.542
	A7	0.97	0.496
Mean SS frequency (Hz) (MODA)	SEED	0.95	0.063
	DOSED	0.93	0.071
	A7	0.77	0.087
Mean KC duration (s) (MASS2-KC)	SEED	0.80	− 0.003
	DOSED	0.64	0.140
	Spinky	0.00	0.671
KC density (epm) (MASS2-KC)	SEED	0.91	0.112
	DOSED	0.91	0.106
	Spinky	0.82	− 0.179
Mean KC PP amplitude (μV) (MASS2-KC)	SEED	0.93	1.157
	DOSED	0.91	3.179
	Spinky	0.88	9.642

SS: sleep spindle; KC: K-complex; epm: events per minute; PP: peak-to-peak. Subject-level parameters correspond to whole-recording aggregates of event-level instances. The correlation is measured between values determined by expert annotations and detections. The difference is defined as the estimated value (by detections) minus the ground truth value (by expert annotations).

**Figure 3:**
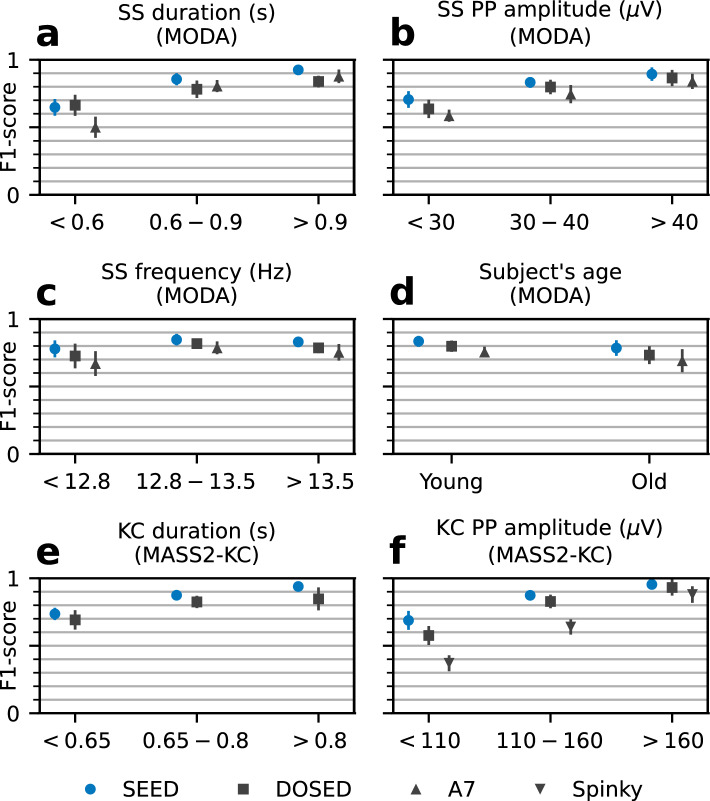
SS and KC detection performance (F1-score) per parameter range. For SSs (from MODA dataset), the parameters considered were (**a**) duration, (**b**) PP amplitude, (**c**) spindle frequency and (**d**) age of the subject. For KCs (from MASS2-KC dataset), the parameters considered were (**e**) duration and (**f**) PP amplitude. Performance is measured by comparing detections and annotations that exist in a given range of the chosen parameter (e.g., between 0.6 s and 0.9 s of duration). Each data point represents the mean ± 2SD of the F1-score computed by micro-average.

To assess the effect of event characteristics on detector performance (e.g., duration and amplitude), F1-scores were obtained for a range of event durations, peak-to-peak (PP) amplitudes, SS frequencies, and ages. Subjects were divided into 2 groups based on age (young and old subjects), whereas the remaining characteristics were divided into consecutive groups of approximately the same number of annotations at the dataset level. Because this arbitrary division caused imbalances at the subject level, micro-average metrics were used for both the MODA and MASS2-KC datasets (see Methods). The results are shown in Fig. 3 (exact F1-scores and test statistics are shown in Supplementary Tables 2 and 3).

SEED outperformed baseline detectors in every comparison except for SSs lasting less than 0.6 s. In this case, the difference in F1-score between SEED and DOSED was non-significant (*p* = 0.18). Further analyses, in this case, revealed that the F1-score was mainly driven by an abnormally high precision (i.e., few false positives) in DOSED’s predictions (the precision was 90.4 ± 2.5% while the recall was 52.6 ± 4.6%). This was a result of DOSED’s tendency to overestimate event duration (see Table 4), which led to few predictions lasting less than 0.6 s. Furthermore, clear associations between event characteristics and detector performance were observed across detectors: Positive associations between F1-scores and both SS and KC duration and PP amplitude; SS detection reliability was higher in the middle range of the SS frequency band; and SS detection accuracy was worse for older subjects as shown by an accuracy reduction of 5% for SEED and 6.4–6.6% for the other detectors.

### Transfer learning analysis

An important application involves using a detector in recordings with potentially different data characteristics compared to the recordings used to develop the detector. A desirable property in this setting is generalization, i.e., the detector’s robustness to changes in the data. However, when the evaluation data distribution differs significantly from the training data distribution, a degradation in performance is expected, unless a correction is made leveraging knowledge of the evaluation data distribution. A common instance of this process for deep learning methods is known as transfer learning, where a detector is *pretrained* (standard training of parameters) on a source dataset and then *fine-tuned* (further training using the previously trained parameters as initialization) on a target dataset. Fine-tuning is expected to require less data than standard training on the target dataset to achieve good performance.

The evaluation data distribution could differ due to changes in the general EEG characteristics (e.g., subject’s demographics), or in the characteristics of expert annotations (e.g., annotation policies). MASS2-SS-E1 and MASS2-SS-E2 have the same EEG signals, and MODA comes from the same cohort (the MASS cohort). However, their annotations show differences in their typical duration and PP amplitude (see Supplementary Fig. 3).

**Figure 4:**
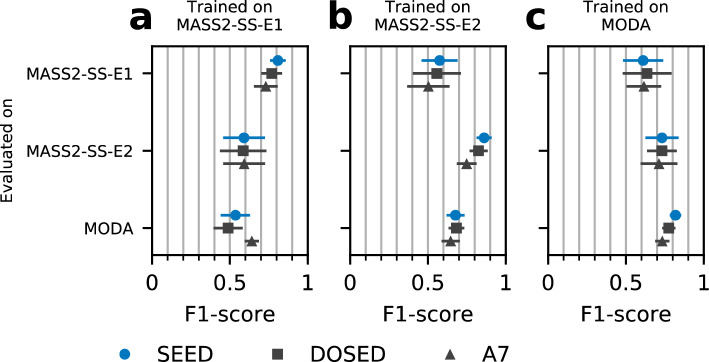
Detector generalization to a dataset not used for training. Performance of SS detection (F1-score) when a detector is trained on one SS dataset and is used directly (without fine-tuning) on another. (**a**) Detectors trained on MASS2-SS-E1. (**b**) Detectors trained on MASS2-SS-E2. (**c**) Detectors trained on MODA. Each data point represents the mean ± 2SD of the F1-score computed by micro-average.

To gauge the degradation in performance that occurs when changing datasets, we trained each SS detector on each SS dataset and we used them to predict directly on the other datasets, without adjustments. The results are shown in Fig. 4 (exact F1-scores and test statistics are shown in Supplementary Table 4). As expected, across transfers, similar performance drop patterns were observed across detectors. In general, the worst transfers involved MASS2-SS-E1, either as the source (recall < 50%) or as the target (precision < 50%).

**Figure 5:**
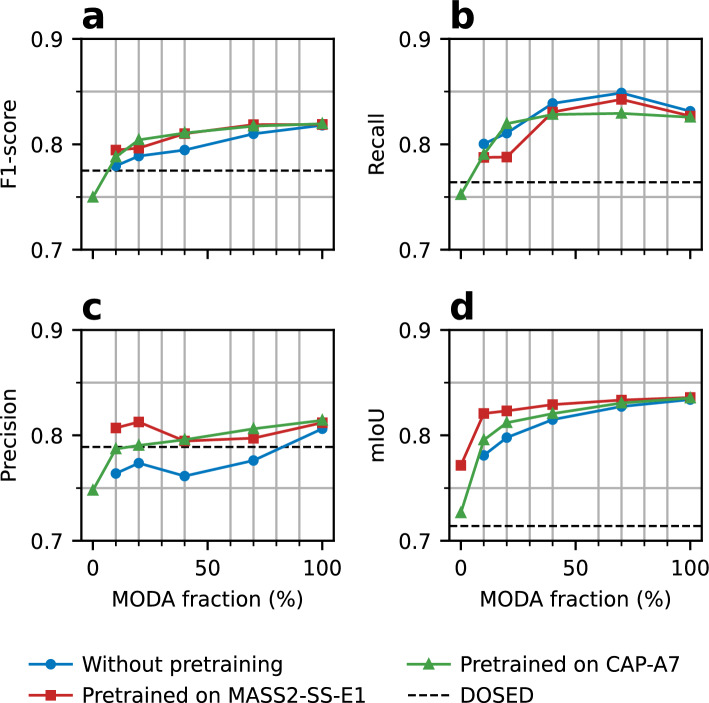
SS detection performance on MODA with fine-tuning after pretraining SEED on another dataset. Fine-tuning is conducted using a fraction of MODA. On the X-axis, a fraction of 0% represents no training, whereas 100% represents no restrictions in size. Shown metrics are (**a**) F1-score, (**b**) recall, (**c**) precision and (**d**) mIoU. Three cases were considered: random initialization and standard training (blue curve); pretraining on the labeled dataset MASS2-SS-E1 (the worst direct transfer, see Fig. 4) and fine-tuning on MODA (red curve); pretraining on the artificial dataset CAP-A7 and fine-tuning on MODA (green curve). The dotted line corresponds to the performance of DOSED trained on the full MODA dataset only. Outliers corresponding to the case pre-trained on MASS2-SS-E1 without fine-tuning (MODA fraction 0%) are not shown in the plots. The performance metrics for this case are F1-score 53.6%, Recall 38.0%, and Precision 92.1%.

Considering the performance drops, we explored fine-tuning for SEED. First, we assessed improvements in the worst transfer performance seen in Fig. 4: SEED was trained from scratch on MASS2-SS-E1 (pretraining) and then retrained on a fraction of MODA (fine-tuning). For comparison, we also evaluated the performance obtained by training on the same fractions of MODA without pretraining. The results of these experiments are shown in Fig. 5 (exact statistics are shown in Supplementary Table 5).

The analyses showed that pretraining SEED on MASS2-SS-E1 allows state-of-the-art performance to be obtained using a small fraction of MODA (F1-score of 79.5 ± 2.4% using 10% of MODA), especially by improving precision (80.7% vs 76.4%, *p* = 0.005, using 10% of MODA) and mIoU (82.1% vs. 78.1%, *p* < 0.001, using 10% of MODA) with respect to the scenario without pretraining. Pretraining had no significant effect on SEED’s performance on MODA when SEED’s training was conducted on the complete MODA dataset. This suggests that the main advantage of pretraining is to reduce the amount of data necessary for achieving high performance with fine-tuning. Indeed, fine-tuning SEED on 10% of MODA was enough to surpass the performance of DOSED shown in Table 2 (*p* = 0.014 for F1-score, *p* < 0.001 for mIoU).

We additionally explored whether pretraining could keep its benefits without requiring expert annotations on the pretraining dataset, further reducing the requirements of labeled data. To that end, SEED was pretrained on the unlabeled dataset CAP using labels generated automatically by the rule-based, handcrafted-feature detector A7 using the parameters reported in the original publication (based on a dataset different from MODA)^21^. This artificial dataset (termed CAP-A7) had 51,597 SS events with a mean SS density of 3.3 events per minute, and a mean SS duration of 1.0 s. During pretraining, SEED reached an F1-score of 88.1 ± 0.3% on CAP-A7. After pretraining on CAP-A7 and fine-tuning on just 10% of MODA, SEED reached an F1-score of 78.8 ± 1.5% on MODA, again surpassing the reference performance of DOSED (*p* = 0.035). The full results of fine-tuning SEED on MODA after pretraining on CAP-A7 are shown in Fig. 5 (and Supplementary Table 5) to ease comparison. Interestingly, across all cases, no significant differences (*p* > 0.18) were observed between F1-score improvements resulting from fine-tuning after pretraining on data with expert-based (MASS2-SS-E1) or rule-based (A7) annotations. However, mIoU saturated faster to a near-maximum when pretraining was conducted on data annotated by experts.

### Population statistics in unlabeled data

Previous research has shown that demographic factors such as age and sex, can influence the characteristics of SSs^11,12,14^. Thus, to evaluate the validity of SEED’s predictions, we examined whether the established associations between demographic factors and SS characteristics were present in SEED's SS detections. For this purpose, SEED was used to generate a large collection of 4,388,910 SS detections from N2 stage EEG signals collected from 11,244 subjects from the unlabeled NSRR6 dataset. As a preliminary validation of this large collection before the main analysis, we verified some desired properties: global statistics (without conditioning on age or sex) for duration, PP amplitude, frequency, and SS density are close to the literature (Supplementary Fig. 5 and Supplementary Table 6); top detections (in estimated probability) are prototypical SSs instead of artifactual noise (Supplementary Fig. 7); and relative sigma power (ratio between average power in the 11–16 Hz and 4.5-30 Hz ranges) correlates with SS density, closely following the ground truth tendency observed in the MODA dataset (Supplementary Fig. 8).

To obtain reliable estimations of the relationships between SS parameters and both age and sex, subjects with less than 10 SS detections (110 subjects, representing 0.98% of the total) were excluded from analyses. Also, for simplicity, 3 subjects aged between 4 and 5 years old were included in the 5-year-old age interval. The resulting dataset included data for both sexes (41% women) and represented the full range of ages between 5 and 90 years old (the age interval with the least subjects was 30–35 years of age, with 15 men and 14 women; the full age and sex distribution is shown in Supplementary Fig. 6).

**Figure 6:**
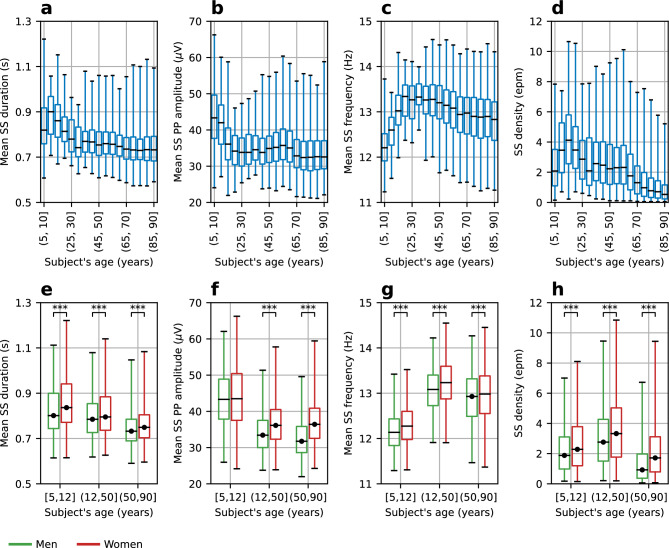
Relationship between SEED’s SS detections, age, and sex on NSRR6. (**a**-**d**) To show age effects, subjects were grouped using 5-year intervals. For each age group, the following SS parameters are shown: (**a**) Mean duration. (**b**) Mean PP amplitude. (**c**) Mean spindle frequency. (**d**) Spindle density. (**e**–**h**) To show sex effects, subjects were grouped using sex and age intervals whose boundaries are the average menarche and menopause ages. For each sex and age group, the following SS parameters are shown: (**e**) Mean duration. (**f**) Mean PP amplitude. (**g**) Mean spindle frequency. (**h**) Spindle density. Women show a greater value than men (*p* < 0.001, indicated by the symbol ***) in every comparison, except for PP amplitude in 5–12 years old (non-significant difference of + 0.6 μV, *p* = 0.29). Black circles at the median indicate data with non-normal distribution. See Methods/Statistics for details on the statistical tests.

The analyses showed that SS duration increases with age, peaks at the age of 10–15 years old (Fig. 6a–d), then progressively decays until the age of 65–70 years old, after which it remains stable. SS amplitude peaks at the age of 5–10 years old, decays strongly until the age of 20–25 years old, remains stable until the age of 60–65 and then decays again at the age of 65–70 years old. SS frequency is minimal at the age of 5–10, increases progressively until age 20–25 years old, remains stable until the age of 40–45 years old, and decays slowly after that. Finally, SS density increases with age and peaks at the age of 15–20 years old, decays strongly until the age of 30–35 years old, remains stable until the age of 55–60 years old, and decays strongly after that. The exploration of the relationship between sex and SS parameters (Fig. 6e–h), showed that women had significantly larger average values than men (*p* < 0.001). The only exception was PP amplitude during childhood (*p* = 0.29). Sex differences were larger for PP amplitude and density during late adulthood. Moreover, unlike men, in women, PP amplitude did not decrease when transitioning from adulthood to late adulthood.

## Discussion

In this work we introduced SEED, a novel deep-learning model for detecting transient events in sleep EEG. Compared to traditional detectors, SEED’s end-to-end approach has the advantage of not requiring handcrafted, rule-based features as it learns to automatically extract relevant features from the data during training. SEED achieved state-of-the-art performance in detecting SSs and KCs. SEED’s architecture features a novel hierarchical design that, by combining convolutional and recurrent layers, allows for the processing of sleep events using long-range contextual information (20 s). This design improved SEED’s capacity to both detect and precisely determine the duration of SS and KC events and enabled it to outperform state-of-the-art detectors in most metrics. Furthermore, differently from previous detectors, SEED does not require arbitrary partitions of the input signals nor fixed-sized sliding or candidate windows, making it simpler to use and more likely to generalize to other transient events with little intervention.

Purcell et al*.*’s pioneering work^14^, applying a rule-based detector (based on wavelet filtering and subsequent thresholding) to a large unlabeled open-source EEG sleep dataset (NSRR6), offered invaluable insight into the interactions between demographic factors and SS characteristics in the population. To validate SEED’s SS performance at the population level, we compared SEED’s SS detections (SEED-SSs) on the NSRR6 dataset with the detections reported by Purcell et al^14^. on the NSRR6 dataset (Purcell-SSs), Warby et al^11^. on the Wisconsin Sleep Cohort—WSC dataset (Warby-SSs), and Lacourse et al^12^. on the MODA dataset (Lacourse-SSs). The main differences among these 4 approaches involve the characteristics of the detectors, post-processing of event duration, and handling of artifacts (for details, see Methods). In general, while SEED replicated the most important results of Purcell-SSs, Warby-SSs, and Lacourse-SSs, the characteristics of SEED-SSs were closer to Warby-SSs and Lacourse-SSs than Purcell-SSs. This suggests that SEED’s detections are closer to the results obtained with the gold standard (expert annotations) than with a rule-based detector. Thus, SEED’s population statistics represent a significant advancement over Purcell et al.’s work, providing a more accurate characterization of SSs in the general population.

The distribution of SS characteristics (duration, PP amplitude, and frequency) in SEED-SSs was similar to the distributions in Purcell-SSs and Warby-SSs. However, SEED-SSs had fewer events with very small PP amplitudes compared to Purcell-SSs and Warby-SSs. In terms of frequency, the SSs detected by the three models were similar in the sigma band, but SEED-SSs and Warby-SSs exhibited events with frequencies up to 16 Hz, whereas events in Purcell-SSs had frequencies up to only 15 Hz. Notably, SEED-SSs included events with frequencies between 10 and 11 Hz, which were absent in Warby-SSs and Purcell-SSs. In Warby-SSs, this difference can be explained by the fact that the WSC dataset does not contain EEG recordings from children, whose spindles typically fall in the lower end of the frequency spectrum. Therefore, 10–11 Hz spindles are expected in a dataset that includes children, such as NSRR6. On the other hand, while Purcell-SSs are based on the same dataset as SEED-SSs (i.e., NSRR6), the detector used by Purcell et al. has limited sensitivity in this frequency range. Regarding the distribution of SS density per subject, Purcell-SSs differed from both Warby-SSs and Lacourse-SSs. Interestingly, the SS density distribution in SEED-SSs was closer to the distributions of Warby-SSs and Lacourse-SSs than to the distribution of Purcell-SSs. As mentioned, this finding suggests that when discrepancies occur between SS characteristics based on rule-based detections (Purcell-SSs) and gold standard expert annotations (Warby-SSs and Lacourse-SSs), SEED’s predictions align with the results obtained using the gold standard method. However, differently from Warby-SSs and Lacourse-SSs, SEED’SSs had a high percentage of cases with 0 events per minute (see Supplementary Fig. 5d). It is likely that this divergence results from the fact that the NSRR6 dataset is biased towards older adults (see Supplementary Fig. 6), who are known to have low sigma activity and, therefore, low spindle density (see Fig. 6d).

SEED’s excellent performance at the population level makes it suitable for establishing population-level reference ranges, which will facilitate the detection of SS and KC biomarkers for diagnostic groups such as schizophrenia and dementia, or SS- and KC-based indices of treatment response in clinical trials^45^. Furthermore, SEED’s minimal requirement for labeled data compared to other detectors, enabled by our novel rule-based pretraining approach, makes our tool a valuable resource for conducting studies in underrepresented or clinical populations for whom labeled data is scarce. This will help reduce some of the biases present in current sleep studies, resulting from limitations related to small sample sizes and unbalanced representation of different age groups, genders, geographic and cultural origins, and clinical populations, among others. In recent years, simpler and wireless alternatives to traditional EEG systems have been developed and validated, thus expanding the population size with access to sleep monitoring^46^. State-of-the-art sleep staging algorithms, such as U-Sleep^47,48^, can further accelerate SS and KC detection by providing SEED with sleep stage information. Collectively, these approaches could pave the way for fully automatic systems with the capacity to accurately detect sleep events in tens of thousands of individuals.

Regarding transfer learning, there were performance drops across the board when the detectors were applied to external, never-seen datasets. Since EEG signals across datasets had similar characteristics, the drops were likely driven by event annotation differences in the training and external datasets. Supplementary Fig. 3 shows the shifts in the distributions of expert annotations for spindle duration and peak-to-peak amplitude across different datasets. Since SS detection on EEG recordings is a segmentation task, such shifts are known to require knowledge of the target distribution to correct the detector^49^. In other words, dealing with this performance drop requires parameter tuning based on the new annotation rules.

Since the shift was mainly in the annotations, there was no need to implement large changes in the feature-extraction and context-integration stages (e.g., layers) of the detectors, just in the final classification stage that implements the criteria used for annotating the events. This fine-tuning process requires little data from the target dataset and was effective in correcting performance.

Pretraining a model on large datasets can improve its generalization, thereby reducing the amount of data needed for fine-tuning on new datasets. The main challenge of pretraining models for biomedical signal analysis is obtaining large amounts of expert-annotated data, which is often difficult to acquire. For this reason, one of the most relevant contributions of our work is demonstrating that pretraining SEED on data (CAP dataset) annotated automatically, without human intervention, using the A7 rule-based algorithm had the same benefits on SEED’s performance on a new dataset as pretraining SEED on expert-annotated data (MASS2 dataset). This suggests that the synthetic annotations generated by a ruled-based algorithm carry enough information about sleep EEG event detection for the model to learn the basic-level features and context relationships necessary to solve the more complex task of emulating the performance of expert raters. This approach is a promising strategy for training deep learning event detectors when there is not enough data for pretraining.

SEED was extensively validated through several experiments including experiments on unlabeled data. The results demonstrated that SEED is a reliable sleep EEG event detector with good generalization to external datasets and results aligned with expert criteria reported in the literature. While SEED outperformed previous detectors on between-subject performance variability, there is still room for improvement, especially in SS detection. SEED’s performance was assessed on healthy subjects older than 5 years old, only. The generalization of SEED’s findings to younger subjects or populations with pathologies such as schizophrenia, will require further investigation.

There are large publicly available sleep EEG datasets that do not have annotations for sleep events, such as those made available by the NSRR. We believe that deep learning detectors would benefit from pretraining on this type of data in combination with synthetic annotations generated by a sleep event detector. An intriguing area for future research would be to investigate the characteristics of synthetic annotations that minimize the need for fine-tuning the detector on the target dataset. The access to large and unbiased population statistics would allow us to detect biases in the population distributions derived from our detector and correct them when necessary. We firmly believe that having open access to large datasets in the order of hundreds of thousands of individuals will become critical to the future development of the field.

## Methods

### Data sources

The algorithms’ performance detecting SSs and KCs was assessed using single-channel, labeled (SS and KC labels) and unlabeled EEG datasets collected during stage N2 at channels C3 or C4. The channels were C3-LE for MASS2, either C3-LE or C3-A2 (depending on the subject) for MODA, and C3-A2 (or C4-A1 if artifactual or unavailable) for CAP and NSRR. Following the format of the MASS2 dataset^35^, data were segmented in 20 s segments. Sleep stage and event (SS and KC) were annotated by expert raters (for details, see Table 1). Each recording used for analyses was collected on a different subject. In cases that had more than one EEG recording, only the first recording was used.

#### MASS2

The Montreal Archive of Sleep Studies (MASS) dataset^35^ is a public whole-night EEG dataset collected on 200 subjects. In this work, we utilized the second subset of MASS (MASS2) comprising annotated EEG recordings segmented in 20 s epochs. The data were collected on 19 subjects (18–33 years old; mean 23.6; SD 3.7). Two experts, E1 and E2, performed SS and KC annotations for stage N2 EEG at channel C3. E1 annotated SSs and KCs for 15 subjects and E2 annotated SSs only for all the subjects. These data were used as 3 distinct EEG datasets: SSs annotated by E1 (MASS2-SS-E1) and E2 (MASS2-SS-E2), and KCs annotated by E1 (MASS2-KC). For simplicity, the 15 subjects annotated by both experts were used for experiments; however, general performance results were obtained using all the subjects in MASS2-SS-E1 and MASS2-KC to facilitate comparisons with the literature.

#### MODA

The Massive Online Data Annotation (MODA) platform was used to generate an open-source dataset with SS annotations made by a consensus of experts on data collected from 180 subjects (18–76 years old; mean 40.6; SD 19.4) from the MASS dataset (MODA dataset)^12^. The MODA dataset is divided into 2 age groups: (1) 100 young subjects (mean age of 24.1 years) annotated by 42 experts, and (2) 80 older subjects (mean age of 62.0 years) annotated by 31 experts. Each expert annotated a sample of 115-s EEG segments collected during stage N2 from electrode C3. In total, MODA consists of 30 subjects with 10 annotated EEG segments each and 150 subjects with 3 annotated segments each. To simplify the analyses, each 115 s segment was extended to 120 s by adding 2.5 s of the signal at the edges, resulting in 6 disjoint 20 s segments.

#### CAP

The Cyclic Alternating Pattern (CAP) sleep dataset^43,44^ is a public dataset consisting of whole-night EEG recordings collected on 108 subjects. CAP’s annotations were conducted on 30 s epochs and include sleep stages only. Subjects with signals with high-frequency noise, non-biological periodic artifacts, or flat sigma activity were excluded from analyses. As a result, N2 data from 80 subjects (14–77 years old; mean 39.5; SD 16.9) collected at channel C4 (C3 if missing) were used for experiments. Epochs with amplitudes > 300μV were discarded.

#### NSRR6

Following Purcell et al.^14^, we consolidated 6 publicly available sleep datasets from the National Sleep Research Resource (NSRR6)^36^: CHAT^37^, CCSHS^38^, CFS^39^, SHHS^40^, MrOS^41^, and SOF^42^. The NSRR6 dataset comprises whole-night recordings collected on 11,630 subjects (40.9% women) aged 4–90 years (mean 58.6; SD 23.5); the recordings have annotations for sleep stages using 30 s epochs. Analyses were conducted on N2 stage data collected with channel C3; C4 data was used when C3 data was either artifactual or not available. We computed statistics on 20 s EEG segments based on the amplitude, standard deviation, and frequency spectrum (fit to a power law distribution), and flagged as artifactual each EEG segment with statistics falling outside the range observed in the MASS2 and MODA datasets. Artifactual signals or subjects with less than 1 h of clean N2 stage data, were excluded from analyses. In total, 96.5% of the subjects and ~ 90% of stage N2 data were kept for analysis.

### Data partition

Labeled datasets (SS or KC annotations) were partitioned with no subject overlap into training, validation, and test sets. We followed a five-fold cross-validation scheme (3 folds for training, 1 for validation, and 1 for testing) in which each subject was used once for testing and once for validation. The scheme was repeated 3 times with different random seeds, resulting in 15 partitions. MODA was stratified according to age group and the number of labeled segments.

MASS2 (n = 15) was used for designing the detector. Eleven subjects were randomly selected and used for network design and hyperparameter search (MASS2-Train); the rest of the subjects (n = 4) were used for testing the detector (MASS2-Test) to ensure an unbiased estimation of performance. Additionally, we conducted a biased evaluation of the detector’s performance using cross-validation with the full MASS2 dataset.

### Performance metrics

#### Metric for individual events

Following Warby et al^11^., the similarity between an annotated event *A* (ground truth) and a prediction *B* was measured using the Intersection over Union (IoU), defined as:

$${\text{IoU}}\left(A,B\right)=\frac{\left|A\cap B\right|}{\left|A\cup B\right|} \in [\mathrm{0,1}],$$

where $\left|A\cap B\right|$ represents the length of the signal segment in which annotation and prediction overlap, and $\left|A\cup B\right|$ represents the length of the overlapping signal segment plus the total length of the non-overlapping segments. Thus, an ${\text{IoU}}\left(A,B\right)$ of 1 (maximum) represents a perfect overlap between prediction and ground truth (*A* = *B*), and an ${\text{IoU}}\left(A,B\right)$ of 0 represents the cases when there is no prediction *B* for an event *A* (undetected event – false negative), or there is no event *A* corresponding to a prediction *B* (false prediction – false positive). For an annotation/prediction pair (*A*, *B*) and a fixed threshold ${\tau }_{{\text{IoU}}}$, a prediction *B* was considered a true positive (TP) when ${\text{IoU}}\left(A,B\right)\ge {\uptau }_{{\text{IoU}}}$; otherwise, *A* was considered a false negative (FN) and *B* a false positive. Thus, TP, FN, and FP are functions of ${\tau }_{{\text{IoU}}}$.

#### Metrics for groups of events

For a given IoU threshold, we computed Recall, Precision, and F1-score. We used ${\tau }_{{\text{IoU}}}=0.2$ to assess the detector’s performance at *finding* events as in previous work^11^. Additionally, we report the mean IoU (mIoU) between valid pairs of events and predictions to assess the detector’s performance at *localizing* events, i.e., at predicting the event’s onset and ending points. That is, a high mIoU indicates a high average temporal overlap between a detection and its associated expert annotation.

#### Development metric

As mentioned above, the F1-score (at ${\tau }_{{\text{IoU}}}=0.2$) captures the performance at detecting events, whereas the mIoU captures the performance at localizing events. However, for model development, i.e., making design decisions and hyperparameter selection, it is convenient to optimize a single metric. For this reason, we capture both performance dimensions simultaneously by measuring the area under the F1-score versus ${\tau }_{{\text{IoU}}}$ curve: high detection performance corresponds to higher F1-score values close to ${\tau }_{{\text{IoU}}}=0$, and high localization performance (high mIoU) has the effect of delaying the decay of the curve (i.e., the F1-score drops after a large threshold). This metric will be referred to as *Average F1-score* (AF1) due to its equivalence to the mean F1-score value calculated across every threshold between 0 and 1. The AF1 allows designing detectors and adjusting hyperparameters without using arbitrary ${\tau }_{{\text{IoU}}}$ values.

#### Metrics for groups of subjects

While a group of subjects can be interpreted as a single group of events, it is often convenient to consider each subject independently. However, the literature is not consistent in this regard. Therefore, to further standardize the framework introduced by Warby et al^11^., we assessed each group of subjects using either a macro-average or micro-average. In a macro-average, each subject contributed equally to the group average. After calculating the metric value of each event, the macro-average was obtained by first collapsing each subject to a single value (e.g., by averaging or computing an F1-score) and then averaging the results of all the subjects. Instead, in a micro-average, each event contributed equally to the group average, so subjects with more events -SSs and KCs- contributed more to the result. The micro-average was obtained by aggregating the values of all the events lumped together, irrespective of the subjects of origin. In general, we used macro-average, except when the dataset or the experiment did not have enough events per subject to allow a reliable estimation of the subject’s metric value. In particular, for MODA we report the micro-average.

#### Metrics in cross-validation

The dataset was partitioned using fivefold cross-validation, without subject overlap between the partitions. To assess a detector’s robustness to changes in the populations or the initialization of the detector, the metrics were computed for each testing set (macro-average) and their means and standard deviations were calculated across testing sets. Instead, to assess a detector’s robustness to individual differences, for each testing dataset, each subject’s metrics were computed (micro-average) and their standard deviation was obtained. This was used as a measure of the dispersion of performance across individuals.

### Overview of SEED’s detection process

The general process is illustrated in Fig. 2a. EEG signals, pre processed using standard procedures, are fed to a deep neural network in 20-s segments. The network then outputs the probability of each sample being part of an event of interest. A threshold ${\uptau }_{p}$ is applied to the probabilities to determine the existence of an event, and a threshold ${\uptau }_{L}<{\uptau }_{p}$ is applied to determine the event’s duration. This process generates a collection of candidate event detections that is further refined with a post-processing specific to the event of interest (SS or KC). Finally, an annotation mask is applied to retain only detections that fall within valid portions of the EEG signals (see “Annotated or selected segments” in Table 1 for the mask choice of each dataset). For example, the annotation mask could be 1 for every sample located in a stage N2 epoch, and 0 elsewhere.

We included EEG segments with non-N2 stages (i.e., with an annotation mask equal to 0) to improve training and inference. During training, EEG segments were randomly extracted with the only restriction that the center must be a valid (N2 stage) sample, to increase robustness to temporal translations. However, since non-N2 samples were masked, these samples were ignored in the calculation of the loss. During inference, we allow the model to make detections on non-N2 samples to consider events located at the borders of N2-stage intervals. However, generated detections that fall completely outside the masked region were ignored.

### EEG preprocessing

Excepting for MODA, EEG signals were bandpass filtered (0.1–35 Hz) with a zero-phase Butterworth filter of order 3. MODA was filtered between 0.3 and 30 Hz to match the original publication’s preprocessing^12^. The signals’ sampling rates were standardized to 200 Hz using a polyphase filtering resampling method, and the signals’ amplitudes were normalized with the standard deviation computed on the training and validation data, but not the testing data. To limit the distorting effect of signal artifacts, for each recording, samples with absolute magnitudes larger than the 99th percentile of the recording’s absolute magnitude distribution, were discarded.

No artifact removal was applied. To avoid extreme amplitudes, normalized signals were clipped at ± 10, which is equivalent to a ± 170 μV range in the non-normalized MASS2-Train dataset.

### SEED’s architecture

SEED’s internal information processing pipeline can be divided into three consecutive stages: local encoding, contextualization, and by-sample classification (see Fig. 2b). SEED’s input consists of EEG signal segments of ${T}_{w}+2{T}_{B}$ samples. The actual EEG window to be scored has a length of ${T}_{w}$ = 4000 samples, but an extra segment of ${T}_{B}$ = 520 samples is attached at each side of the window to avoid border effects (for details see below). SEED outputs a by-sample probability vector (dense segmentation) of length ${T}_{w}/8$, equivalent to one event prediction for every 8 input samples. In general terms, the local encoding stage uses a 1D convolutional block that extracts local features from the input signal and downsamples it by a factor of 8, transforming it in a multivariate time series of length ${T}_{w}/8$. Next, the contextualization stage uses a sequential block to integrate features extracted from distant, non-neighboring samples. Finally, the by-sample classification stage takes the temporally contextualized data and uses a 1D convolutional layer and a softmax function to assign each EEG sample the probability of being part of an event (positive class) or background signal (negative class). The output’s temporal resolution is 8 times lower than the temporal resolution of the original EEG signal. Thus, for EEG signals sampled at 200 Hz (5 ms interval between consecutive samples), SEED outputs a prediction every 40 ms of the input signal. We consider this time span dense enough for detecting SSs and KCs which usually have durations of at least 300 ms. While this reduced temporal resolution was used to compute the loss function, at inference time, SEED’s output time series is linearly upsampled to match the sampling rate of the input EEG signals.

The architectures of SEED’s stages are illustrated in Fig. 2b. In the beginning, a batch normalization (batchnorm) layer^50^ is applied to improve normalization and introduce small-scale noise during training. The local encoding stage comprises a series of two 1D convolutional layers followed by two convolutional multi-dilated blocks (Conv MDBs). The Conv MDB is a custom block consisting of four 2-layered convolutional blocks processing information in parallel, each with a different dilation rate in its kernel (see Fig. 2c). This design allows for an increase in Conv MDBs’ receptive fields without increasing the number of parameters or compromising their capacity to extract granular information. Convolutional layers used throughout SEED have a kernel size of 3, zero-padding, and are followed by batchnorm and Rectified Linear Unit (ReLU)^51^ layers. Pooling layers (average pooling) with a size of 2 were used after each block for subsampling. The number of channels at the first two 1D convolutional layers is *F* = 64, which doubles after each subsampling step, reaching a total of 4*F* = 256 channels at the last Conv MDB of the local encoding stage. The receptive field (EEG segment length) underlying each time point of the local encoding stage’s output was 204 time points (1.02 s). To reduce border effects caused by zero-padding, 0.6 s worth of input EEG signal were removed from each border of the encoding stage’s output. The contextualization stage comprises two Bi-directional Long Short-Term Memory (BLSTM) layers, each with ${N}_{1}$=256 neurons per direction, followed by a 1D convolutional layer with a kernel size of 1, ${N}_{2}$=128 channels, and a ReLU layer. Long Short-Term Memory (LSTM)^52^ layers are a common type of recurrent layer with memory; BLSTMs concatenate two independent LSTM layers, one traversing the input from left to right, and the other from right to left. We applied dropout^53^, a regularization method that randomly replaces output values of the preceding layer with zeros, to each layer in this stage with dropout probabilities ${\rho }_{1}$, ${\rho }_{2}$ and ${\rho }_{2}$, respectively (${\rho }_{1}$=0.2, ${\rho }_{2}$=0.5). To ensure a minimum bidirectional context at the contextualization stage (see Fig. 2b), we cropped 2 s from each border after the last BLSTM layer. Considering both border requirements, we set ${T}_{B}$=520 (2.6 s). Hyperparameters were set by optimizing AF1 on the MASS2-Train subset. Supplementary Table 7shows the evaluated options and selected values of the hyperparameters.

### Training

Weights were initialized following standard practices^54,55^, except for the output neuron’s bias for the positive class. Following previous work on segmentation neural networks, the bias was initialized to ${\text{log}}\left({p}_{1}/\left(1-{p}_{1}\right)\right)$, with ${p}_{1}$=0.1 being a small positive class probability^56^. This decision avoids training instabilities resulting from the loss function being dominated by the background EEG class.

Attached to the EEG signal we have two binary time series of the same length, one representing expert annotations and the other the annotation mask (e.g., a mask indicating stage N2 segments). We randomly sample M = 32 points from EEG signals (having a positive mask). Each point is used as the central point of a 20-s window, making up a batch of 32 training examples. Each window includes the time series of the EEG signal, labels, and mask. The random center selection allows the model to increase its robustness to temporal translations. A training epoch was defined as the number of iterations that traverse the entire collection of disjoint 20-s segments with a positive mask.

Compared to background activity, SSs and KCs are rare events. To generate a balanced training batch, M/2 center points were extracted from segments exhibiting high event activity, while the remaining M/2 center points were extracted from segments displaying low event activity. The boundary between high and low event activity was determined by the median event activity of the entire training dataset.

### Loss function

Let the binary label vector $\mathbf{y}\in {\{\mathrm{0,1}\}}^{{T}_{w}}$ be the annotation mask of the training segment $\mathbf{m}\in {\{\mathrm{0,1}\}}^{{T}_{w}}$, and let $\mathbf{p}\in {{\text{R}}}^{\left({T}_{w}/8\right)\times 2}$ be the probabilities of the predicted classes. To match the model’s classification rate (1 prediction every 8 samples), the labels (**y**) and masks (**m**) were downsampled using an average pooling layer of size 8 followed by rounding the values to the nearest integer. If the *k*-th sample is of class ${y}_{k}$, then the corresponding class probability ${p}_{k}\left({y}_{k}\right)$ should be maximum. To achieve this, we minimized the segment’s weighted cross-entropy loss, in which each sample’s weight included its mask value (${m}_{k}$) and a class-specific weight (${w}_{class}\left({y}_{k}\right)$). Therefore, the loss was set as follows:

$${\text{l}}\left(\mathbf{y},\mathbf{p}\right)=-\frac{{\sum }_{k=1}^{{T}_{w}/8}{m}_{k}{w}_{class}\left({y}_{k}\right){\text{log}}{p}_{k}\left({y}_{k}\right)}{{\sum }_{k=1}^{{T}_{w}/8}{m}_{k}{w}_{class}\left({y}_{k}\right)}$$

### Data augmentation

Some transformations that should not change the labels were applied to the input signal during training. First, we applied additive noise uniformly sampled from [− 1, 1] μV. Next, we either increased or decreased the activity in some frequency bands in ways that do not change the labels according to expert knowledge. For SSs, 4-8 Hz and 7–10 Hz activity was increased in the background EEG and decreased inside events, and 0.5–2 Hz activity was increased or decreased anywhere. For KCs, 11–16 Hz activity was increased or decreased anywhere. Let **x** be an EEG segment of $T={T}_{w}+2{T}_{B}$ samples (more than 20 s). To decrease activity in a given frequency band $\left({f}_{1},{f}_{2}\right)$, **x** was replaced with $\mathbf{x}- a\mathbf{w}\mathbf{z}$, where $a$ is a scale factor (uniformly sampled from [0, 1]), $\mathbf{z}={\text{BPF}}\left[{f}_{1},{f}_{2}\right]\left(\mathbf{x}\right)$ is the result of bandpass filtering the signal **x** between frequencies ${f}_{1}$ and ${f}_{2}$, and **w** is a window that leaves only a random portion of **z**, with random center and duration uniformly sampled from [1, 5] s. Furthermore, to increase activity, **x** was replaced with $\mathbf{x}+\mathbf{w}\mathbf{z}$, where $\mathbf{z}\left({f}_{1},{f}_{2},{A}_{max}\right)$ is a randomly generated oscillatory signal, with a variable frequency between ${f}_{1}$ and ${f}_{2}$ and a variable amplitude between 0 and ${A}_{max}$, and **w** is a window generated using the procedure described above. To avoid experimental overhead, the hyperparameter ${A}_{max}$ was set according to the amplitude statistics of MASS2-Train, using E1 annotations, leading to 18μV for 0.5–2 Hz, 20 μV for 4–8 Hz, 12 μV for 7–10 Hz, and 10 μV for 11–16 Hz.

### Optimization

The Adam optimizer^57^ was used for loss minimization with a learning rate of ${10}^{-4}$, default exponential decay rates, and a batch size of 32. The validation AF1 (using ${\uptau }_{p}$=0.5) was computed after each training epoch for assessing model quality. If the model did not improve for 5 consecutive training epochs, the learning rate was decayed by a factor of 2 and the count was restarted. Training was stopped, and the best model was selected if 200 epochs were reached or no improvement was observed after 4 decays. As recommended for recurrent architectures^58^, the maximum norm of the gradient was limited to 1.

### Fine-tuning

The fine-tuning of pretrained models followed the same optimization procedures described above but with an initial learning rate of ${5\cdot 10}^{-5}$ and a maximum of 3 decays instead of 4. This is equivalent to skipping to the first decay in a regular training.

### Inference probability vector

The model processes entire EEG recordings using windows of length ${T}_{w}$=4000 (20 s) and steps of length ${T}_{w}/2$. For each predicting window, only the central half was kept. These segments were concatenated to generate a probability time series covering the complete EEG recording.

### Collection of detections

To generate detections (i.e., event onset and ending timestamps), the probability time series was first linearly upsampled by 8 to match the input signal sampling frequency. Next, inspired by traditional detectors, we used two thresholds: ${\uptau }_{p}$ (for existence) and ${\uptau }_{L}<{\uptau }_{p}$ (for duration). Let $p\in \left(\mathrm{0,1}\right)$ be the output probability for the positive class. Given a detection threshold ${\tau }_{p}$, *p* was transformed to an *adjusted probability* $\widetilde{p}\in \left(\mathrm{0,1}\right)$ defined as:

$$\widetilde{{\text{z}}}={\text{log}}\left(\frac{{\text{p}}}{1-{\text{p}}}\right)-{\text{log}}\left(\frac{{\uptau }_{{\text{p}}}}{1-{\uptau }_{{\text{p}}}}\right),$$

$$\widetilde{{\text{p}}}=\frac{1}{1+{\text{exp}}\left(-\widetilde{{\text{z}}}\right)}.$$

This implies that $\widetilde{p}$ has a detection threshold of 0.5, recovering the common practice of selecting the most likely class. This transformation also simplifies ensembling models since $\widetilde{p}$ has the same interpretation in every model. Then, a detection was defined as an interval having $\widetilde{p}\ge {\uptau }_{L}$ and at least one sample satisfying $\widetilde{p}\ge 0.5$. A post-processing step can be applied to this collection of detections to integrate domain knowledge. Finally, only detections with at least one sample inside the annotation mask were kept.

The threshold ${\tau }_{p}$ was set at the end of training by grid search to maximize AF1 in the combination of the training and validation sets. We found that using the validation set alone increased the variance of the predictions. For simplicity, we fixed ${\uptau }_{L}=0.85\cdot 0.5=0.425$. Using two thresholds resulted in better performance compared to using only ${\tau }_{p}$.

### Event probability

The model outputs probabilities for individual samples, making event probabilities (i.e., probabilities for intervals) ambiguous. For analysis purposes, we defined the probability of an event as the 75th percentile of its adjusted probabilities. We used the 3^rd^ quartile instead of the median or the mean to gain robustness against the event borders, which typically have low probabilities.

### Post-processing for sleep spindle detections

Following standard procedures^5^, detections closer than ${\Delta }_{{\text{sep}}}$ were combined, and detections shorter than ${\Delta }_{min}$ were removed. Given that SS duration distribution is mostly bounded^14^, detections longer than ${2\Delta }_{max}$ were discarded, and the ${\Delta }_{max}$ in the center were kept for detections lasting between ${\Delta }_{max}$ and ${2\Delta }_{max}$. Following Warby et al.^11^, the intervals were set to ${\Delta }_{{\text{sep}}}$=0.3 s, ${\Delta }_{min}$=0.3 s and ${\Delta }_{max}$=3 s.

### Post-processing for K-complex detections

Considering that KCs have a duration of at least 0.5s^5^, predictions shorter than 0.3 s were removed, which allows some room for prediction error. Multiple KCs might be predicted as a single event due to the lack of minimum separation, while they are considered disjoint in MASS2-KC. To split combined detections, we proposed a new post-processing, inspired by the negative peak detection of traditional detectors like Spinky^25^. Within each prediction, the lowpass filtered signal with 4 Hz cut-off was used to find negative peaks. To avoid border artifacts, peaks located at less than 0.05 s from the start of the prediction or less than 0.2 s from the end of the prediction were ignored. Next, peaks without a zero-crossing in between were averaged, so that each KC candidate was represented by a single negative peak. If more than one peak remained after this procedure, the prediction was split at the middle point between the peaks. After splitting, detections shorter than 0.3 s were removed.

### Baselines with open-source training implementation

SEED’s performance was compared to the performance of 3 open-source detectors from the literature: DOSED^16^, A7^21^, and Spinky^25^. DOSED is a convolutional neural network method that predicts SSs and KCs on 20-s EEG segments. A7 is a signal processing method that predicts SSs using a sliding window of 0.3 s to compute features that are combined using expert rules. Spinky is a signal processing method that predicts SSs and KCs by decomposing signals into oscillatory and transient components, and then applying simple detection rules. We did not use Spinky to predict SSs due to its poor performance. Additionally, Spinky only provides the location of KCs’ negative peaks, so we assumed that KCs start 0.1 s before and end 1.3 s after their peaks, as it is done in its original publication.

Grid searches were conducted to adjust Spinky’s and A7’s thresholds to maximize AF1 in the training set. After training, DOSED’s output threshold was adjusted using the same procedure as for SEED. The remaining hyperparameters matched those reported in the original publications.

### Baselines without open-source training implementation

Three additional deep learning methods were found in the literature that did not offer a training implementation: SpindleNet^22^, a convolutional and recurrent neural network method that predicts SSs on small 0.25-s windows; SpindleU-Net^17^, a convolutional neural network method that predicts SSs on 20-s windows by returning a dense segmentation of the signal (a class score for each time step); and the method proposed in Lechat et al^14^., abbreviated here as DKL-KC, a fully-connected neural network method that predicts KCs on candidate 6-s windows. Similar to Spinky, DKL-KC only provides the location of KCs.

For these detectors, we used their reported cross-validation performance on MASS2 using the experimental settings closest to our approach. For SpindleNet, we used the reported performance against the union of E1 and E2 annotation sets as the performance against E2, since the union of experts is approximately the same as E2 alone. For SpindleU-Net, we use the reported performance against each expert on the whole dataset (19 subjects for E1 and 15 subjects for E2). The original publication reports the standard deviation between subjects instead of partitions, which would be an unfair comparison since the dispersion between subjects tends to be larger. However, it also reports the performance for each subject. Based on this, we built subsets of subjects matching our cross-validation partitions and took their average value as a way to simulate each partitions’ performance, which allowed us to estimate a standard deviation between such partitions. For DKL-KC, we used the reported performance on the whole dataset (19 subjects). Both SpindleNet and SpindleU-Net were evaluated with a post-processing that considered a different minimum duration. While SEED, DOSED and A7 were evaluated using 0.3 s, SpindleNet was evaluated using 0.4 s and SpindleU-Net using 0.5 s in their original publications.

### Subject-level metrics in the MODA dataset

Some of the analyses require computing a metric for individual subjects instead of the whole dataset at once (e.g., between-subject variability and subject-level SS parameters). While most datasets in this work have enough EEG data per subject to obtain reliable metrics, it is not the case for MODA. With the purpose of obtaining reliable metrics for the MODA dataset for subject-level analyses, metric calculations in that type of experiments were conducted on the subsample of subjects (n = 28) who had at least 19 min of EEG data and at least 10 SS annotations.

### Normalization of EEG signals in transfer learning

The input normalization required for SEED and DOSED, was conducted separately using the source and target datasets as references. The best performance was achieved when using statistics from the source dataset. For DOSED, this involved scaling the standardized signals by the ratio between the standard deviations of the target and source datasets.

### Perturbation transformations on EEG signals

For the perturbation analysis (see Supplementary Fig. 4), we considered three types of perturbations: scaling, axis inversion, and band-stop filtering. Scaling consisted in multiplying the EEG signal by a factor between 0.5 and 1.5. Axis inversion included both Y and X axis inversions; for Y axis inversions signals were multiplied by -1 (amplitude inversion) and for the X axis inversions, signals were reversed in time (time inversion). Finally, band-stop filtering consisted of applying a band-stop filter to the EEG signal to remove a specific frequency band.

### SS detections on the unlabeled NSRR6 dataset

The detections were obtained by averaging the probability outputs of the 5 SEED models, which were obtained using five-fold cross-validation on MODA. NSRR6 signal’s preprocessing and SEED’s detection post-processing matched those used for MODA. In addition, detections with duration > 3 s or with PP amplitudes > 134.12 μV (maximum amplitude in MODA) were rejected to reduce the effects of artifacts (< 0.1% of detections were rejected).

### Methodological differences with respect to literature in the NSRR6 dataset

In addition to the differences between detectors, our approach diverges from Purcell et al^14^. in the post-processing of event duration and the handling of artifacts. Purcell et al.’s post-processing discarded SSs lasting less than 0.5 s and merged SSs closer than 1 s. Instead, we followed the criteria proposed by Warby et al^11^., which allows shorter and closer SSs. Moreover, Purcell et al. removed artifacts from EEG signals following several criteria: arousals, movements, aberrant segments according to power analysis, anomaly detection according to signal statistics, cardiac interference correction, among others. In contrast, we relied on the processing capabilities of deep learning approaches which allowed using minimal preprocessing. EEG segments with statistics (from amplitude and power spectrum) outside the range seen on MASS signals, were discarded; this approach allowed us to double the data used by Purcell et al. (36,548 h vs. 16,499 h of N2 sleep).

### Statistics

For each experiment, data distributions were tested for normality using the Kolmogorov–Smirnov test. For normally distributed data, differences between means (e.g., between the F1-score of SEED and a baseline detector) were assessed using Welch's unequal variances t-test. For non-normally distributed data (see Table 3 footnote and Fig. 6 caption), mean differences were assessed using the non-parametric Mann–Whitney U test. All tests were two-sided with a significance level of 0.05.

### Supplementary Information

Supplementary Information.

## Data Availability

All datasets are publicly available. The polysomnography data for the MASS2 and MODA datasets are available from http://ceams-carsm.ca/mass/. The expert annotations for the MODA dataset are available from https://osf.io/8bma7/wiki/home/. The polysomnography data for the CAP dataset is available from https://physionet.org/content/capslpdb/1.0.0/. The polysomnography data for the NSRR6 dataset is available from the National Sleep Research Resource website https://sleepdata.org/datasets.

## Code availability

The code and pretrained weights are publicly available at https://github.com/nicolasigor/Sleep-EEG-Event-Detector. SEED was implemented using TensorFlow 1.14 in Python 3.7.
