# Supplementary material for: A robust deep learning detector for sleep spindles and K-complexes: towards population norms
Source: Sci Rep. 2024 Jan 2;14:263. doi: 10.1038/s41598-023-50736-7 (PMC10762090; doi:10.1038/s41598-023-50736-7)
Supplement: Supplementary file 1 — Supplementary Information. [file 41598_2023_50736_MOESM1_ESM.pdf]

A Robust Deep Learning Detector for Sleep Spindles and K-Complexes: Towards Population Norms

Nicolás I. Tapia-Rivas[1]

Pablo A. Estévez[1, 2, 3] *

José A. Cortes-Briones[4, 5, 6]

[1] Department of Electrical Engineering, University of Chile, Santiago, Chile

[2] Millennium Institute of Intelligent Healthcare Engineering, Santiago, Chile

[3] IMPACT, Center of Interventional Medicine for Precision and Advanced Cellular Therapy, Santiago, Chile

[4] Schizophrenia and Neuropharmacology Research Group at Yale (SNRGY), Department of Psychiatry, Yale University School of Medicine, New Haven, CT, USA

[5] Abraham Ribicoff Research Facilities, Connecticut Mental Health Center, New Haven, CT, USA

[6] VA Connecticut Healthcare System, West Haven, CT, USA

* Email: pestevez@cec.uchile.cl

SUPPLEMENTARY INFORMATION

Estimation of SEED's design bias on the MASS2 dataset

To investigate the existence of a possible design bias in SEED (i.e., an over-optimistic result driven by an architecture particularly suitable for MASS2 signals), we assessed the performance of SEED, DOSED, A7, and Spinky on MASS2-Test, on 4 subjects that were kept out of SEED's development process (see Data partition in Methods). Instead of applying a cross-validation scheme where both validation and testing subsets alternated cyclically (the scheme used for performance comparison in Table 2), the testing subset was fixed to MASS2-Test.

The mean F1-score and mIoU in Supplementary Table 1, are similar to those in Table 2, as is the difference in performance between detectors, indicating negligible design bias. The standard deviations are different (and smaller) than those in Table 2, which is expected due to the testing set being fixed (and thus not exhibiting inter-subject variability).

Supplementary Table 1: SS and KC detection performance on MASS2-Test.

Dataset	Detector	F1-score (%)		mIoU (%)	
		Mean ± SD	p-value	Mean ± SD	p-value
MASS2-SS-E1	**SEED**	81.0 ± 0.5		85.1 ± 0.2	
	DOSED	78.0 ± 0.5	<0.001	75.3 ± 1.3	<0.001
	A7	69.7 ± 0.4	<0.001	74.9 ± 0.2	<0.001
MASS2-SS-E2	**SEED**	85.1 ± 0.5		78.0 ± 0.2	
	DOSED	81.8 ± 0.7	<0.001	73.8 ± 0.5	<0.001
	A7	73.4 ± 0.1	<0.001	74.8 ± 0.1	<0.001
MASS2-KC	**SEED**	83.3 ± 0.4		90.5 ± 0.2	
	DOSED	78.0 ± 0.9	<0.001	72.2 ± 1.3	<0.001
	Spinky	65.7 ± 0.2	<0.001	42.3 ± 0.1	<0.001

mIoU: mean Intersection over Union; N.A.: not available. MASS2-Test is a subset of 4 subjects that were kept out of the entire development process of SEED (see Data Partition in Methods). Metrics of SEED (proposed detector), DOSED, A7 and Spinky were obtained using open-source implementations. P-values are defined against SEED's performance.

Correlation between experts and detectors for subject-level parameters of SSs and KCs

Table 4 presents statistics for the relationship between expert-derived and detector-derived subject-level parameters. These relationships are represented with scatter plots in Supplementary Figure 1 for SS detection and in Supplementary Figure 2 for KC detection. It can be observed that a linear relationship is an accurate approximation of the relationship observed in the samples, especially for SEED.

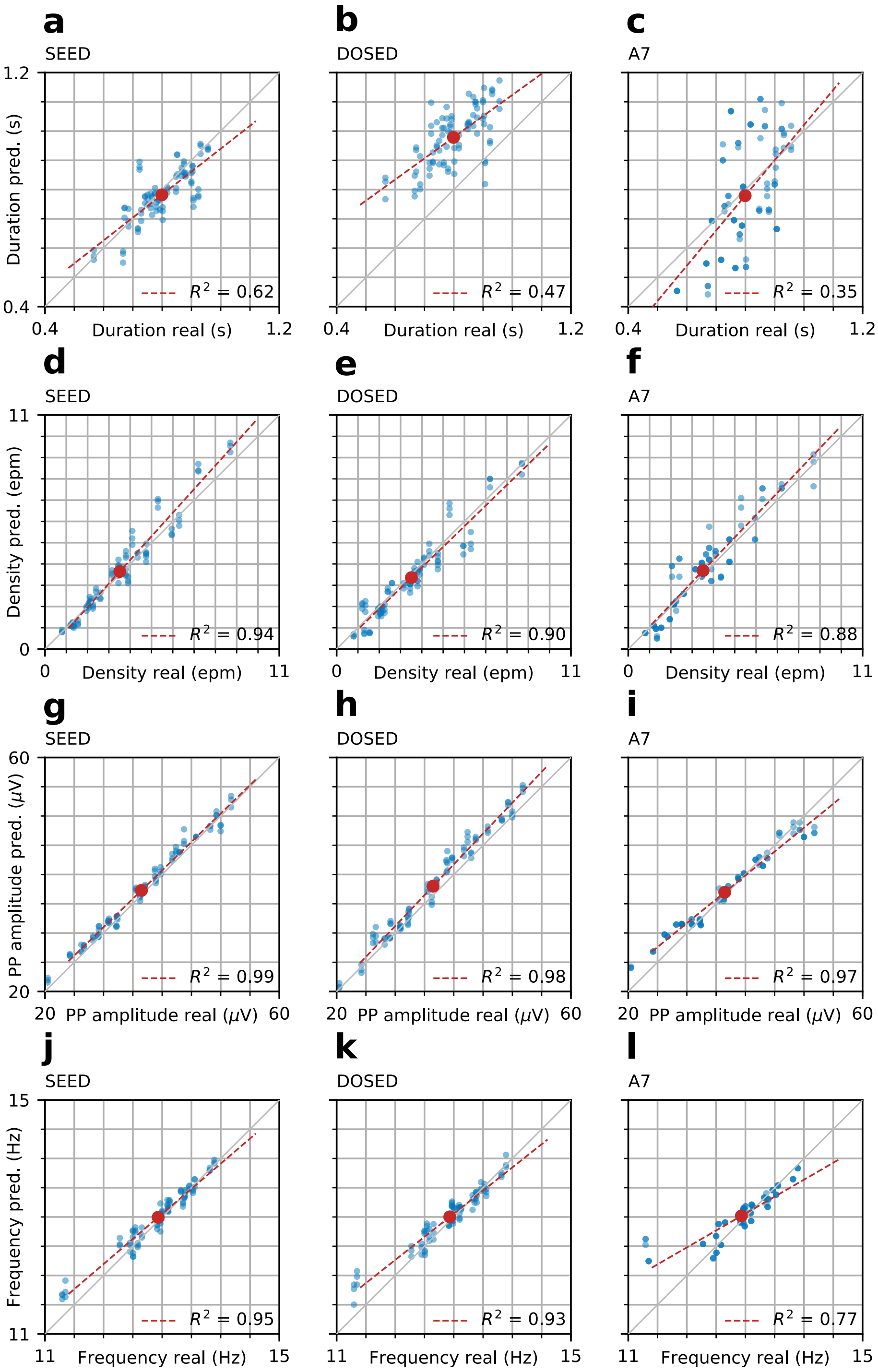

Supplementary Figure 1: Correlation between experts and detectors for subject-level parameters of SSs. Each panel presents a scatter plot for the MODA dataset, comparing the real value (from expert annotations) against the predicted value from detections. The parameters considered were (a-c) mean duration, (d-f) density, (g-i) mean PP amplitude, and (j-l) mean SS frequency.

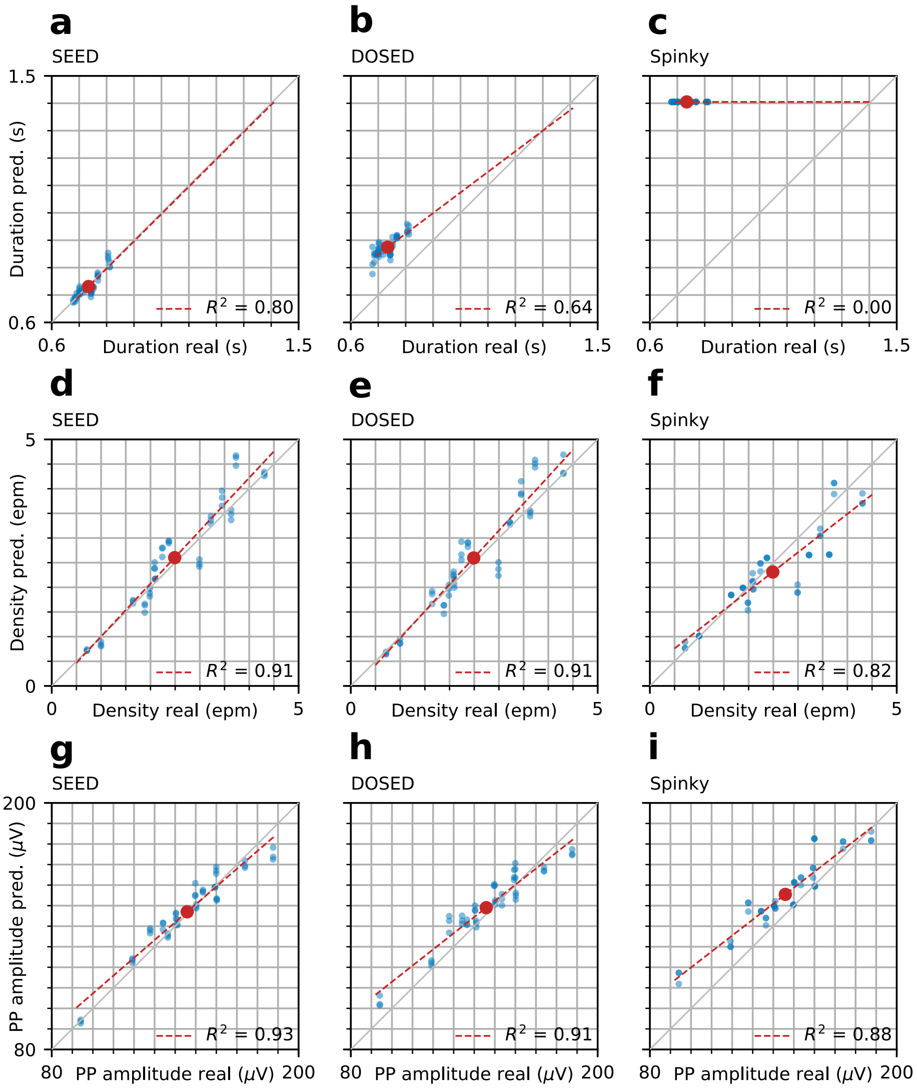

Supplementary Figure 2: Correlation between experts and detectors for subject-level parameters of KCs. Each panel presents a scatter plot for the MASS2-KC dataset, comparing the real value (from expert annotations) against the predicted value from detections. The parameters considered were (a-c) mean duration, (d-f) density, and (g-i) mean PP amplitude.

SS and KC detection performance per parameter range

Figure 3 illustrates the effect of parameter ranges on SS and KC detection performance. The exact F1-scores, along with p-values for the comparison against SEED, are shown in Supplementary Table 2 for SS detection and in Supplementary Table 3 for KC detection.

Supplementary Table 2: SS detection performance (F1-score) per parameter range.

Parameter	Range	F1-score (%)				
		SEED	DOSED		A7	
		Mean ± SD	Mean ± SD	p-value	Mean ± SD	p-value
SS duration (s) (MODA)	<0.6	64.8 ± 2.6	66.3 ± 3.4	0.178	50.0 ± 3.4	<0.001
	0.6 – 0.9	85.6 ± 1.7	78.2 ± 2.7	<0.001	80.5 ± 1.7	<0.001
	>0.9	92.5 ± 1.0	83.9 ± 1.6	<0.001	87.8 ± 2.0	<0.001
SS PP amplitude (μV) (MODA)	<30	70.6 ± 2.6	63.6 ± 2.9	<0.001	58.6 ± 1.7	<0.001
	30 – 40	83.3 ± 1.5	79.8 ± 2.2	<0.001	74.6 ± 2.9	<0.001
	>40	89.4 ± 2.0	86.5 ± 2.5	0.002	84.0 ± 2.3	<0.001
SS frequency (Hz) (MODA)	<12.8	77.9 ± 2.6	72.6 ± 4.0	<0.001	67.0 ± 4.1	<0.001
	12.8 – 13.5	84.6 ± 1.6	81.7 ± 1.1	<0.001	78.7 ± 1.9	<0.001
	>13.5	83.0 ± 1.2	78.6 ± 1.5	<0.001	75.3 ± 2.6	<0.001
Subject's age (MODA)	Younger	83.5 ± 1.5	79.8 ± 1.6	<0.001	75.5 ± 1.5	<0.001
	Older	78.5 ± 2.4	73.2 ± 2.8	<0.001	69.1 ± 3.8	<0.001

SS: sleep spindle; epm: events per minute; PP: peak-to-peak. The performance is computed by restricting the collection of annotations and detections to have parameter values within a given range. P-values are defined against SEED's performance.

Supplementary Table 3: KC detection performance (F1-score) per parameter range.

Parameter	Range	F1-score (%)				
		SEED	DOSED		Spinky	
		Mean ± SD	Mean ± SD	p-value	Mean ± SD	p-value
KC duration (s) (MASS2-KC)	<0.65	73.6 ± 1.8	69.1 ± 3.1	<0.001	70.0 ± 3.6	0.003
	0.65 – 0.8	87.4 ± 1.2	82.4 ± 1.8	<0.001	73.3 ± 1.7	<0.001
	>0.8	93.9 ± 1.2	84.7 ± 3.8	<0.001	56.0 ± 7.4	<0.001
KC PP amplitude (μV) (MASS2-KC)	<110	68.8 ± 3.0	57.6 ± 3.0	<0.001	37.0 ± 2.5	<0.001
	110 – 160	87.3 ± 1.2	82.8 ± 2.0	<0.001	63.9 ± 2.3	<0.001
	>160	95.4 ± 1.3	93.1 ± 2.5	0.006	87.9 ± 2.6	<0.001

KC: K-complex; epm: events per minute; PP: peak-to-peak. The performance is computed by restricting the collection of annotations and detections to have parameter values within a given range. P-values are defined against SEED's performance.

Transfer learning

Supplementary Figure 3 illustrates the distribution shifts found in expert annotations when considering different labeled SS datasets. The SS annotations found in MASS2-SS-E1, unlike those in other datasets, tend to have a duration closer to the mean duration, with practically no annotations lasting less than 0.5s. On the other hand, these annotations tend to have a larger amplitude and almost no annotations exhibiting less than 20μV PP amplitude.

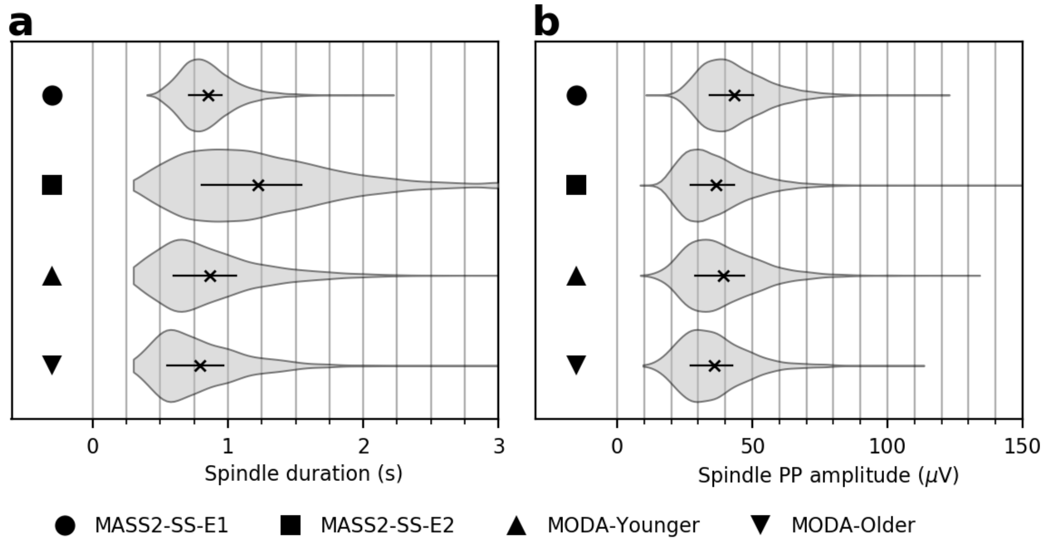

Supplementary Figure 3: SS parameters distribution for expert annotations. To illustrate the difference in signal characteristics of expert annotations determined by different experts, each panel compares the distribution of event-level SS parameters between labeled datasets, where MODA was further divided into two subsets: MODA-Younger (100 subjects with mean age 24.1) and MODA-Older (80 subjects with a mean age 62.0). (a) Distributions of the duration of each SS. (b) Distributions of the PP amplitude of each SS. In each distribution, we include the mean (indicated by a cross) and the interquartile range (indicated by a solid line).

Figure 4 shows the SS detection performance drops that occur when using a trained detector on a dataset not used for training and without fine-tuning. The exact F1-scores and p-values for the comparisons against SEED are shown in Supplementary Table 4.

Supplementary Table 4: Detector generalization to a dataset not used for training.

Training dataset	Evaluation dataset	F1-score (%)				
		SEED	DOSED		A7	
		Mean ± SD	Mean ± SD	p-value	Mean ± SD	p-value
MASS2-SS-E1	MASS2-SS-E1	80.8 ± 2.1	76.8 ± 2.9	<0.001	73.0 ± 3.4	<0.001
	MASS2-SS-E2	59.1 ± 6.3	58.5 ± 7.1	0.823	59.1 ± 6.3	0.974
	MODA	53.6 ± 4.3	48.9 ± 4.3	0.006	64.1 ± 1.8	<0.001
MASS2-SS-E2	MASS2-SS-E1	57.5 ± 5.3	55.7 ± 7.3	0.461	50.3 ± 6.4	0.002
	MASS2-SS-E2	86.1 ± 2.0	82.5 ± 2.5	<0.001	74.9 ± 2.8	<0.001
	MODA	67.7 ± 2.5	68.3 ± 2.1	0.452	64.6 ± 2.5	0.002
MODA	MASS2-SS-E1	61.1 ± 6.0	63.6 ± 7.4	0.318	61.5 ± 5.2	0.821
	MASS2-SS-E2	73.2 ± 4.9	73.1 ± 4.4	0.961	71.2 ± 5.5	0.317
	MODA	81.8 ± 1.4	77.5 ± 1.7	<0.001	73.3 ± 1.9	<0.001

The performance is computed by training the detector in the training dataset and evaluating the detector, without any fine-tuning, in the evaluation dataset. P-values are defined against SEED's performance.

Figure 5 shows how SEED's performance improves with fine-tuning and the effect of different types of pretraining (including no pretraining at all). The exact statistics of F1-score, Recall, Precision, and mIoU are shown in Supplementary Table 5.

Supplementary Table 5: SS detection performance on MODA with fine-tuning after pretraining SEED on another dataset.

Fraction of MODA (%)	Pretraining dataset	F1-score (%)	Recall (%)	Precision (%)	mIoU (%)
0	MASS2-SS-E1	53.6 ± 4.3	38.0 ± 4.5	92.1 ± 1.5	77.2 ± 1.0
	CAP-A7	75.0 ± 1.5	75.2 ± 1.9	74.8 ± 2.5	72.7 ± 0.8
10	None	77.9 ± 1.5	80.0 ± 4.6	76.4 ± 4.1	78.1 ± 1.5
	MASS2-SS-E1	79.5 ± 2.4	78.8 ± 6.0	80.7 ± 3.6	82.1 ± 0.8
	CAP-A7	78.8 ± 1.5	79.0 ± 2.9	78.7 ± 2.7	79.6 ± 1.2
20	None	78.9 ± 1.6	81.1 ± 5.2	77.4 ± 4.8	79.8 ± 0.9
	MASS2-SS-E1	79.6 ± 2.0	78.8 ± 6.7	81.3 ± 4.5	82.3 ± 0.8
	CAP-A7	80.4 ± 1.1	82.0 ± 2.3	79.0 ± 2.4	81.2 ± 0.6
40	None	79.5 ± 2.6	83.9 ± 6.9	76.1 ± 4.3	81.5 ± 1.0
	MASS2-SS-E1	81.0 ± 1.9	83.1 ± 5.4	79.5 ± 3.3	82.9 ± 0.5
	CAP-A7	81.1 ± 1.3	82.8 ± 2.9	79.6 ± 3.4	82.1 ± 1.0
70	None	81.0 ± 1.5	84.9 ± 2.9	77.6 ± 3.2	82.7 ± 0.5
	MASS2-SS-E1	81.9 ± 1.3	84.3 ± 2.8	79.7 ± 2.7	83.3 ± 0.5
	CAP-A7	81.7 ± 1.1	82.9 ± 2.3	80.6 ± 1.9	83.1 ± 0.6
100	None	81.8 ± 1.4	83.1 ± 2.7	80.6 ± 2.5	83.4 ± 0.5
	MASS2-SS-E1	81.9 ± 1.3	82.7 ± 2.7	81.2 ± 2.1	83.6 ± 0.5
	CAP-A7	81.9 ± 1.2	82.6 ± 2.2	81.4 ± 2.4	83.5 ± 0.5

SS: sleep spindle. The performance is obtained by pretraining SEED on the pretraining dataset and then fine-tuning on a fraction of the MODA dataset. A fraction of 0% represents no training, whereas 100% represents no restrictions in size.

Perturbation analysis of SEED

Experiments were conducted to assess SEED's sensitivity to perturbation in the EEG using input scaling, axis inversion, and band-stop filtering in SS and KC detection tasks. The changes in F1-score after each perturbation are shown in Supplementary Figure 4.

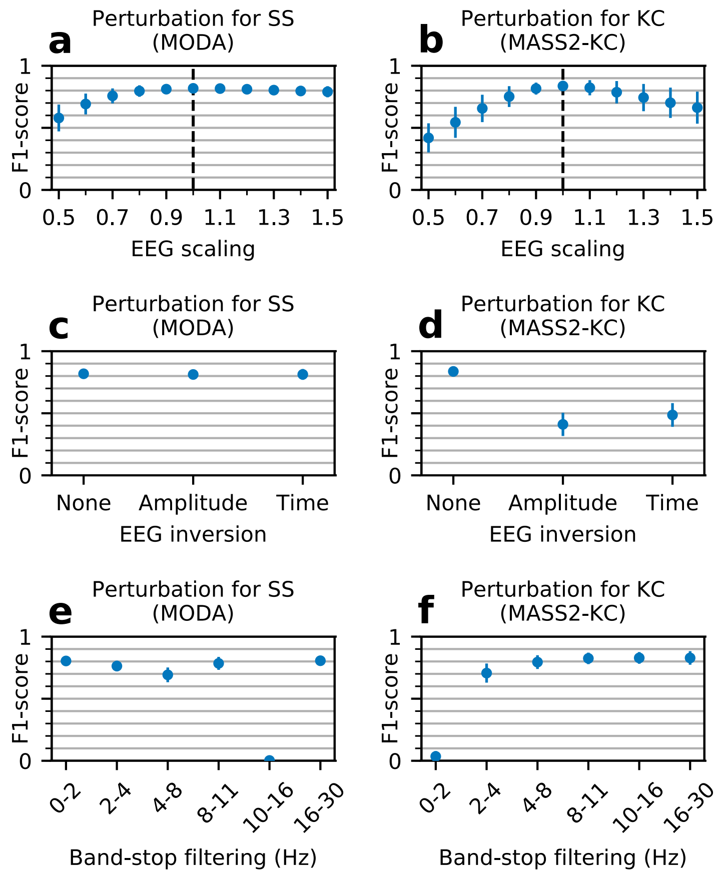

Supplementary Figure 4: Perturbation analysis. Three types of perturbations were used. (a-b) Changing the amplitude of the EEG by multiplying the signal with a scale factor between 0.5 and 1. (c-d) Inverting the EEG by changing the sign of its values (amplitude inversion) or by reversing the temporal axis (time inversion). (e-f) Band-stop filtering to remove a specific frequency band. Each data point represents the mean ± 2SD of the F1-score computed by micro-average.

The scaling perturbation had a similar effect on both SS and KC detection. As expected, recall and precision changed in opposite directions in response to scaling; higher amplitude led to more detections implying that the amplitude of EEG signals is a relevant feature in deciding the existence of an event. The analyses revealed that signal amplitude was slightly more relevant for KC than for SS detection due to the higher sensitivity of the F1-score to the scaling factor. Furthermore, amplitude perturbation had little effect on mIoU, suggesting that event duration is not affected by global scaling.

Inversion perturbation had different effects on SS and KC detection. SS detection was mostly unaffected by inversions, which is consistent with the fact that SSs are mostly symmetrical both in amplitude and time. In contrast, both types of inversion caused large drops in KC detection performance, which is consistent with the fact that KCs are asymmetrical in both amplitude and time.

The band-pass filtering perturbation also affected SS and KC detection differently. For SS detection, filtering out components below 2Hz had no significant effects on performance; between 4-8Hz, it substantially reduced precision; and between 10-16Hz, it reduced performance to zero. In accordance with the literature, these results suggest that SEED's detections rely on activity in the sigma frequency band, which is complemented by information from the 4-8Hz band that is used to discard false positives.

For KC detection, filtering out frequency components below 4Hz reduced performance to zero; between 4-8Hz, it substantially reduced precision; and above 8Hz, it had no effect on performance. In line with the literature, these results suggest that SEED's detections rely on the activity below 4Hz, complemented by information extracted from the 4-8Hz band that is used to discard false positives.

General SS statistics in NSRR6

SEED was used to generate a large collection of 4,388,910 SS detections from N2 stage EEG signals obtained from 11,244 subjects from the unlabeled NSRR6 dataset. This large collection allows robust statistics to be obtained for the most common parameters of SSs. The distributions of three event-level parameters (duration, peak-to-peak amplitude, and frequency) and one subject-level parameter (density of SSs during N2 stage) are shown in Supplementary Figure 5. Central and variation measurements of these distributions are shown in Supplementary Table 6.

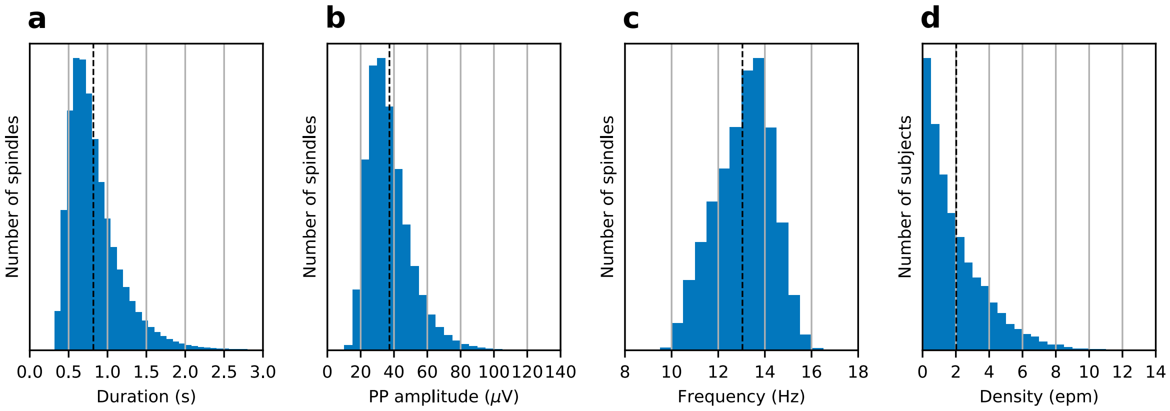

Supplementary Figure 5: Distribution of SS parameters on NSRR6 according to SEED's detections. (a-c) Event-level parameters: (a) duration, (b) PP amplitude, and (c) spindle frequency. (d) Subject-level density. The average of each distribution is indicated by a dotted vertical line.

Supplementary Table 6: SS parameters statistics on NSRR6 according to SEED's detections.

Parameter	Mean	Central 50% interval	Central 90% interval
Duration (s)	0.82	0.56 – 0.96	0.44 – 1.48
PP amplitude (μV)	37.3	27.7 – 43.8	20.6 – 62.9
Frequency (Hz)	13.0	12.2 – 13.9	10.9 – 14.9
Density (epm)	2.02	0.60 – 2.94	0.14 – 5.84

SS: sleep spindle; PP: peak-to-peak; epm: events per minute. Duration, PP amplitude, and frequency are event-level parameters, whereas density is a subject-level parameter. The central 50% and 90% intervals correspond to the range determined by the percentiles 25 to 75 and 5 to 95, respectively.

The NSRR6 dataset has thousands of subjects, covering a large range of sex and age demographics. However, the coverage is not uniform. To interpret the previous statistics in the right context, Supplementary Figure 6 shows the age distribution for each sex. Older adults represent most of the dataset. The statistics shown in Supplementary Figure 5 and Supplementary Table 6 are largely influenced by this population.

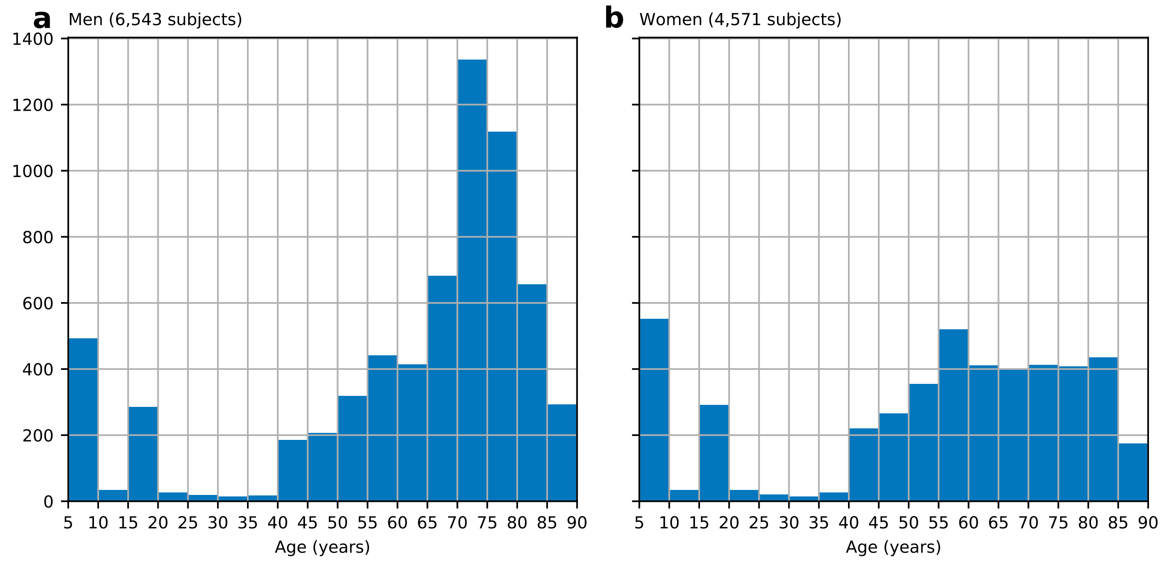

Supplementary Figure 6: Subject's demographics on NSRR6. (a) Age distribution of men. (b) Age distribution of women. Only subjects with at least 10 SEED's detections of SSs are shown.

Prototypical SSs in NSRR6 according to SEED

In Supplementary Figure 7, five SSs were assigned the highest detection probability by SEED (0.99 event probability). These detections can be interpreted as prototypes of "ideal" SSs, as learned by SEED during training. The prototypes have a clear spindle-like shape (i.e., a waxing and waning shape) that stands out from the background signal, an abrupt onset and offset, large amplitude, a duration of ~1s, and little activity outside the sigma band (11-16Hz) except for some slow waves (0-2Hz). Furthermore, EEG signals in the SSs' immediate context are free from artifactual contamination in the 11-16Hz frequency range, and they occur in the context of other high-probability SSs.

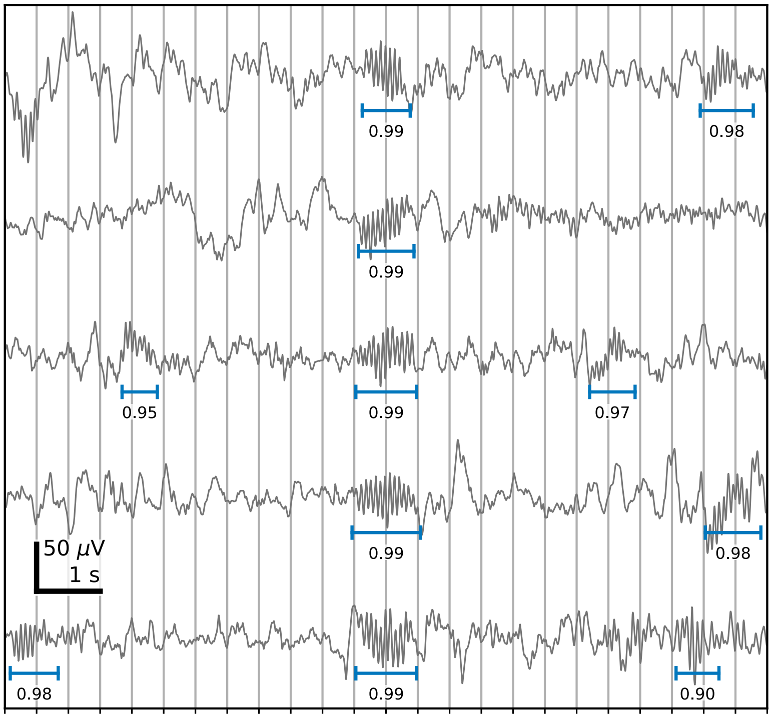

Supplementary Figure 7: Examples of 5 EEG segments with "ideal" SS detections on the NSRR6 dataset. The spindles at the center of each EEG segment were detected by SEED with a probability of 0.99. Other SS detections can be seen in other segments of the signals. All SS detections are indicated by horizontal blue lines, with their event probabilities annotated below.

Relationship between sigma power and SS density

Relative sigma power (ratio between average power in the 11-16Hz and 4.5-30Hz ranges) is expected to correlate with SS density in sleep studies[1]. Analyses revealed that the relationship between NSRR6's relative sigma power and SS density, derived from SEED's SS detections, shows a pattern similar to the one observed in the relationship between relative sigma power and SS density derived from expert annotation for the MODA dataset. This can be interpreted as a ground truth for this relationship (Supplementary Figure 8).

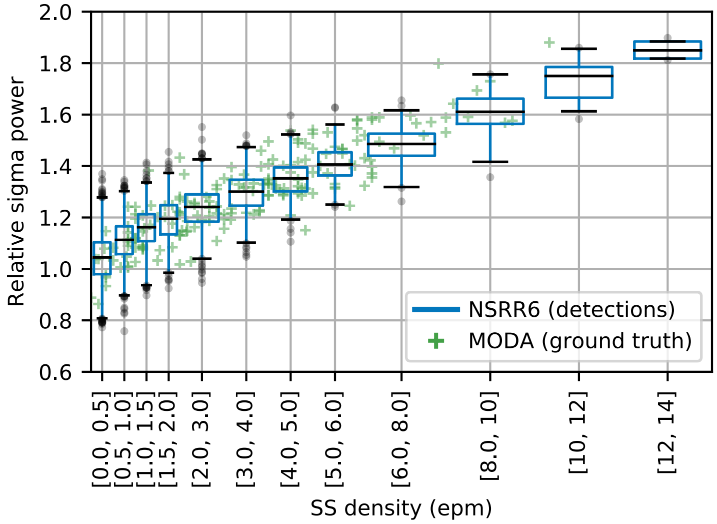

Supplementary Figure 8: Relationship between relative sigma power and SS density in SEED's detections on NSSR's stage N2 data. The relationship between SEED's SS detection density on NSRR6 (unlabeled for SSs) and relative sigma power (ratio between average power in the 11-16Hz and 4.5-30Hz ranges) is shown with blue boxplots. The relationship between SS density (expert labels) and relative sigma power on the MODA dataset is shown with green markers as ground truth, for comparison purposes.

Selected hyperparameter and design decisions

Hyperparameters and architectural decisions of the neural network model were selected through experiments on the MASS2-Train subset of the data. In each experiment, performance was measured using the proposed AF1 metric with cross-validation (see Methods for details), and the options with the highest AF1 were kept.

For the neural network architecture, we explored different options for both the local encoding stage and the contextualization stage to arrive at our selected design shown in Figure 2b. For the local encoding stage, we stacked convolutional layers, starting with blocks made of two layers followed by subsampling, and we tested several variations including dilated layers, blocks with residual connections, parallel branches concatenated at the end, and varying numbers of blocks and layers within a block. For the contextualization stage, we explored recurrent architectures (LSTM, GRU[2], and their bidirectional versions), convolutional architectures (like temporal convolutional networks[3] and encoding-decoding convolutional networks[4]), and self-attention architectures[5]. We found that convolutional alternatives reached a lower mIoU, and self-attention alternatives required pre-training to reach RNNs' performance, without additional gains.

For the selected components, the value of each hyperparameter was selected through experiments. Supplementary Table 7 details the selected value and evaluated options of each hyperparameter. The choice of 20s for the input signal is mainly based on the literature (previous detectors and the MASS2 pagination). To make an experimentally informed decision, we explored other window sizes, from 5s to 120s, and we found that performance saturated at 15-23s windows, so we kept 20s for simplicity.

Supplementary Table 7: Summary of hyperparameter values.

Hyperparameter	Selected Value	Evaluated Options
Extra EEG samples at borders (T_B)	520	Determined by the signal that is removed at the borders after convolutions (0.6s) and BLSTM (2s)
Input EEG signal length	20s	5s to 120s
Initial convolutional filters (F)	64	16, 32, 64, 128
Type of pooling layer	Average pooling	Average pooling or Max pooling
Number of convolutional blocks	3	1 to 5
Size of BLSTM layers (N_1)	256 per direction	64, 128, 256, 512 per direction
Size of last hidden layer (N_2)	128	32, 64, 128, 256
Dropout rate after local encoding stage (ρ_1)	0.2	0, 0.2, 0.5
Dropout rate after BLSTM (ρ_2)	0.5	0, 0.2, 0.5
Training batch size	32	16, 32, 64
Initial learning rate	10^{-4}	$10^{-2}, 10^{-3}, 10^{-4}, 10^{-5}, 10^{-6}$
Learning rate decay factor	2	2 or 10
Learning rate decay patience	5 epochs	5 or 10 epochs

REFERENCES

1. Purcell, S. M. *et al.* Characterizing sleep spindles in 11,630 individuals from the National Sleep Research Resource. *Nat Commun* **8**, 15930 (2017).
2. Cho, K. *et al.* Learning Phrase Representations using RNN Encoder–Decoder for Statistical Machine Translation. in *Proceedings of the 2014 Conference on Empirical Methods in Natural Language Processing (EMNLP)* 1724–1734 (2014).
3. Bai, S., Kolter, J. Z. & Koltun, V. An empirical evaluation of generic convolutional and recurrent networks for sequence modeling. *arXiv preprint arXiv:1803.01271* (2018).
4. Ronneberger, O., Fischer, P. & Brox, T. U-Net: Convolutional Networks for Biomedical Image Segmentation. in *Medical Image Computing and Computer-Assisted Intervention – MICCAI 2015* 234–241 (2015).
5. Vaswani, A. *et al.* Attention is all you need. in *Advances in Neural Information Processing Systems 30* 5998–6008 (2017).
